# CERAMIC: Case-Control Association Testing in Samples with Related Individuals, Based on Retrospective Mixed Model Analysis with Adjustment for Covariates

**DOI:** 10.1371/journal.pgen.1006329

**Published:** 2016-10-03

**Authors:** Sheng Zhong, Duo Jiang, Mary Sara McPeek

**Affiliations:** 1 Department of Statistics, University of Chicago, Chicago, Illinois, United States of America; 2 Department of Human Genetics, University of Chicago, Chicago, Illinois, United States of America; University of Oxford, UNITED KINGDOM

## Abstract

We consider the problem of genetic association testing of a binary trait in a sample that contains related individuals, where we adjust for relevant covariates and allow for missing data. We propose CERAMIC, an estimating equation approach that can be viewed as a hybrid of logistic regression and linear mixed-effects model (LMM) approaches. CERAMIC extends the recently proposed CARAT method to allow samples with related individuals and to incorporate partially missing data. In simulations, we show that CERAMIC outperforms existing LMM and generalized LMM approaches, maintaining high power and correct type 1 error across a wider range of scenarios. CERAMIC results in a particularly large power increase over existing methods when the sample includes related individuals with some missing data (e.g., when some individuals with phenotype and covariate information have missing genotype), because CERAMIC is able to make use of the relationship information to incorporate partially missing data in the analysis while correcting for dependence. Because CERAMIC is based on a retrospective analysis, it is robust to misspecification of the phenotype model, resulting in better control of type 1 error and higher power than that of prospective methods, such as GMMAT, when the phenotype model is misspecified. CERAMIC is computationally efficient for genomewide analysis in samples of related individuals of almost any configuration, including small families, unrelated individuals and even large, complex pedigrees. We apply CERAMIC to data on type 2 diabetes (T2D) from the Framingham Heart Study. In a genome scan, 9 of the 10 smallest CERAMIC p-values occur in or near either known T2D susceptibility loci or plausible candidates, verifying that CERAMIC is able to home in on the important loci in a genome scan.

## Author Summary

Case-control association testing has proven to be useful for identification of genetic variants that affect susceptibility to disease. One can expect to gain power for detecting such variants by including relevant covariates in the analysis, by accounting for any relatedness of sampled individuals, and by making use of partial information in the data. For analysis of continuously-varying traits, variations on linear mixed-model (LMM) approaches have proven effective at achieving some of these goals. However, for case-control or binary trait mapping, there remain significant challenges. Direct application of LMM approaches to binary traits suffers from power loss when covariate effects are strong, and existing generalized LMM approaches can perform poorly in the presence of trait model misspecification and partially missing data. We propose CERAMIC, a method for binary trait mapping, which is computationally feasible for large genome-wide studies, and which gains power over previous approaches by improved trait modeling, retrospective assessment of significance, accounting for sample structure, and making use of partially missing data. We illustrate this approach in genome-wide association mapping of type 2 diabetes in data from the Framingham Heart Study.

## Introduction

In genetic association analysis of a binary trait, such as presence or absence of a disease, it can be useful to properly control for relevant covariates. Inclusion of environmental risk factors in the analysis has the potential to increase statistical power by reducing phenotypic noise, and adjustment for confounding factors can provide some protection against spurious association [1, 2]. For a sample of independent individuals, logistic regression provides a natural approach. However, it is common for association studies to contain some related individuals. This can arise in isolated populations, or by chance in large samples in outbred populations, or, for example, when families collected for linkage studies are included in association analysis. In the presence of related individuals, it is important to model the familial correlation in the sample appropriately to avoid inflation of the significance of association [3] and to improve power [4]. Furthermore, if missing data occur in samples with related individuals (for instance, when some individuals are phenotyped but not genotyped or vice versa), additional power can often be obtained by appropriately incorporating partial information [5].

It is of interest to develop an association mapping method for binary traits that addresses the aforementioned challenges, yet has computational time complexity feasible for current scales of genome-wide association studies (GWASs). The classical transmission disequilibrium test (TDT) [6] and the FBAT method [7] are applicable only to family-based designs. For general case-control designs with distantly-related individuals, methods [8–10] based on linear mixed models (LMM) are commonly used in recent literature. However, such methods are specifically designed for quantitative traits, and, although they can be applied to case-control data, they can suffer from power loss due to failure to capture the binary nature of the outcome. More recent work [11, 12] specifically addresses the analysis of ascertained case-control samples with low levels of relatedness. To model binary traits in samples with high levels of relatedness, several authors [13–15] have proposed methods based on the generalized linear mixed model (GLMM) statistical framework, which extends the classical logistic regression model by including genetic random effects on the logit scale. Of these, only GMMAT [15] has a computational speed that is feasible for large data sets. For samples with unknown population structure, Jiang et al. [16] recently proposed CARAT, a binary-trait genome-wide association testing approach, which adjusts for relevant covariates on a logistic, instead of linear, scale, and which incorporates an additive polygenic effect within a computationally efficient quasi-likelihood framework. However, none of the above methods exploit information contained in partially missing data. MQLS [4] is a binary-trait association testing method that allows related individuals in the sample and that makes full use of the relationship information in order to incorporate partially missing data, but it does not adjust for covariates or for additive polygenic effects. MASTOR, [17] a more recent association method for samples with related individuals, is able to use information in partially missing data by applying a retrospective analysis to a LMM. Like other LMM approaches, it is designed for quantitative traits, although it can be applied to binary traits.

We consider a somewhat different setting from that addressed by CARAT and GMMAT. We assume a sample that includes at least some closely-related individuals, for whom the pedigree structure is assumed known. We also want to allow for the possibility of incorporating partially missing data, which is not possible with CARAT or GMMAT. We propose CERAMIC (Case-control Efficient Related-individual Association Mapping Incorporating Covariates), a mixed-model, binary-trait association testing method, which adjusts for relevant covariates and efficiently incorporates missing data to enhance power. The mean and covariance structure of CERAMIC is tailored for binary traits, and it would therefore be expected to outperform LMM methods such as EMMAX, [8] GEMMA [10] and MASTOR. By using a quasi-likelihood framework, CERAMIC achieves computational efficiency for genome-wide analysis. When assessing significance, we modify the genotypic model used by CARAT to allow for possible correlation between the genotype and the covariates, which is of particular relevance when, for example, ancestry-informative covariates are included to control for population stratification. As a further development over CARAT and a further advantage over prospective LMM and GLMM methods (e.g. GMMAT, EMMAX, GEMMA), CERAMIC effectively exploits partially missing data to improve power by incorporating data on individuals with missing genotypes who have a genotyped relative. Our method can also be considered a generalization of MQLS to allow adjustment for covariates and additive polygenic effects. Unlike the family-based, covariate-adjusted TDT test, CERAMIC can be applied to completely general samples of related and unrelated individuals, provided the genealogy is known.

Major features of CERAMIC are summarized as follows: (1) it is applicable to essentially arbitrary combinations of related and unrelated individuals, including small outbred pedigrees and unrelated individuals, as well as large, complex inbred pedigrees; (2) it incorporates information on individuals with partially missing data while correctly accounting for dependence; (3) it corrects binary phenotypes for both covariates and additive polygenic effects using a model that exploits the binary nature of the trait; and (4) it is computationally feasible for current association studies. For comparison, we have developed two other retrospective tests that also have features (1), (2) and (4) above, but which do not model additive polygenic effects: MQLS-LOG, which uses a logistic approach to adjust binary phenotypes only for covariates, not for polygenic effects, and MQLS-LIN, which uses an ordinary least-squares regression approach to do the same. We conduct simulation studies to assess the type 1 error and power of our methods, CERAMIC, MQLS-LOG and MQLS-LIN, and to compare them to previously reported methods, MASTOR, EMMAX, GEMMA, CARAT and GMMAT. Finally, we apply CERAMIC to the analysis of type 2 diabetes (T2D) data from the Framingham Heart Study (FHS).

## Materials and Methods

We consider a binary trait measured on a sample of *n* individuals. Denote the phenotype by a vector, ***Y***, of length *n*, where the *i*th element, *Y*_*i*_, is the value of the phenotype for individual *i*. Suppose we also observe *k* − 1 ≥ 0 covariates, encoded in a design matrix, ***X***, of dimension *n* × *k*, where an intercept (i.e., a column of ones) is always included in ***X*** in addition to the *k* − 1 non-trivial covariates. Let ***X***_*i*_ denote the column vector of covariate values for individual *i* (so that $X_{i}^{T}$ is the *i*th row of the matrix ***X***). Let ***G*** denote the vector of genotypes for the *n* individuals at a tested biallelic variant, where *G*_*i*_ = 0, 1, or 2 denotes minor allele count of the *i*th individual at the variant. In the following sub-sections, we first briefly review the CARAT method before presenting our CERAMIC approach.

### A brief overview of CARAT

CARAT [16] was developed for binary trait association mapping in samples that are possibly subject to unknown population structure, assuming that genome-wide data are available. CARAT is based on a quasi-likelihood model in which only the conditional mean and variance of ***Y*** given genotype and covariate information are specified. The conditional mean is given by

$$\text{E}\left(Y_{i}|X,G\right)=\mu_{i},\;\;\text{with}\;\;\log\frac{\mu_{i}}{1-\mu_{i}}=X_{i}\beta +G_{i}\gamma ,\;\;i=1,\cdots ,n,$$ (1)

where ***β*** is a *k*-dimensional vector of unknown covariate effects, and *γ* is the unknown scalar association parameter. The conditional variance is given by

$$\Omega :=\text{Var}\left(Y|X,G\right)=\Gamma^{1/2}\Sigma \Gamma^{1/2},\text{with}\;\;\Sigma =\xi \Phi +\left(1-\xi \right)I,$$ (2)

where **Γ** is an *n* × *n* diagonal matrix with *i*th diagonal element equal to *μ*_*i*_(1 − *μ*_*i*_), ***I*** is an *n* × *n* identity matrix, **Φ** is a genetic relationship matrix, and 0 ≤ *ξ* ≤ 1 is an unknown scalar parameter measuring the relative importance of additive polygenic vs. i.i.d. error variance in explaining trait variability. The appearance of the terms **Γ**^1/2^ in the formula for **Ω** in Eq (2) ensures that, for an outbred individual *i*, the relationship between the conditional mean and variance given by Var(*Y*_*i*_|***X***, ***G***) = *μ*_*i*_(1 − *μ*_*i*_) holds, as is necessary for a binary random variable. In CARAT, **Φ** is taken to be an empirical genetic relationship matrix calculated from genome-wide data. The unknown parameter for CARAT is (*γ*, ***β***, *ξ*), where, for association analysis, *γ* is the main parameter of interest, while (***β***, *ξ*) is typically considered a nuisance parameter.

To detect association between the phenotype and the SNP, *H*_0_: *γ* = 0 is tested against *H*_1_: *γ* ≠ 0. To form the CARAT test statistic, we first obtain the null estimate, $\left(0,{\hat{\beta }}_{0},{\hat{\xi }}_{0}\right)$ of the parameter (*γ*, ***β***, *ξ*) by iteratively solving a system of estimating equations under the constraint *γ* = 0. The quasi-score estimating equation for ***β*** is given by

$$X^{T}\Gamma^{1/2}\Sigma^{-1}\Gamma^{-1/2}\left(Y-\mu \right)=0,$$ (3)

where ***μ*** = ***μ***(*γ*, ***β***) = (*μ*_1_, ⋯, *μ*_*n*_)^*T*^. The estimating equation for *ξ* is given by

$${\left(Y-\mu \right)}^{T}\Gamma^{-1/2}\Sigma^{-1}\left(\Phi -I\right)\Sigma^{-1}\Gamma^{-1/2}\left(Y-\mu \right)=\text{trace}\left(\Sigma^{-1}\left(\Phi -I\right)\right).$$ (4)

Then $\left({\hat{\beta }}_{0},{\hat{\xi }}_{0}\right)$ is defined to be the solution to the system consisting of Eqs (3) and (4), with *γ* constrained to be 0. The CARAT test is based on the quasi-score statistic,

$$U_{0}≔G^{T}{\hat{\Gamma }}_{0}^{1/2}{\hat{\Sigma }}_{0}^{-1}{\hat{\Gamma }}_{0}^{-1/2}\left(Y-{\hat{\mu }}_{0}\right),$$ (5)

where ${\hat{\mu }}_{0}$, ${\hat{\Sigma }}_{0}$ and ${\hat{\Gamma }}_{0}$ are ***μ***, **Σ** and **Γ** evaluated at $\left(\gamma ,\beta ,\xi )=(0,{\hat{\beta }}_{0},{\hat{\xi }}_{0}\right)$. (For additional details on quasi-score tests and their use in statistical genetics see references [18] and [19]).

To assess the significance of the association test, CARAT takes a retrospective approach, in which it is assumed that, under the null hypothesis of no association,

$${\text{E}}_{0}\left(G|X,Y\right)=2p1_{n}\;\;\text{and}\;{\text{Var}}_{0}\left(G|X,Y\right)=\sigma_{g}^{2}\Phi ,$$ (6)

where 0 ≤ *p* ≤ 1 is the unknown allele frequency of the variant of interest, $\sigma_{g}^{2}>0$ is an unknown parameter, and the subscript “0” indicates that expectation and variance are taken under the null hypothesis. CARAT estimates $\sigma_{g}^{2}$ using ${\tilde{\sigma }}_{g}^{2}=2\hat{p}(1-\hat{p})$, where $\hat{p}=0.5\times \bar{G}$ is the sample average estimator of *p*. The CARAT test statistic can then be defined by

$$\text{CARAT}≔\frac{{\left(Z^{T}G\right)}^{2}}{{\tilde{\sigma }}_{g}^{2}\cdot Z^{T}\Phi Z},\;\;\text{where}\;Z={\hat{\Gamma }}_{0}^{1/2}{\hat{\Sigma }}_{0}^{-1}{\hat{\Gamma }}_{0}^{-1/2}\left(Y-{\hat{\mu }}_{0}\right).$$ (7)

Significance of association is assessed by comparing the test statistic to a $\chi_{1}^{2}$ random variable.

In addition to testing for association, one can also obtain an estimate of the full parameter (*γ*, ***β***, *ξ*) by solving a system consisting of Eqs (3) and (4) and the following quasi-score estimating equation for *γ*:

$$G^{T}\Gamma^{1/2}\Sigma^{-1}\Gamma^{-1/2}\left(Y-\mu \right)=0.$$ (8)

The system consisting of Eqs (3), (4) and (8) can be solved iteratively to obtain the estimated parameter vector.

### CERAMIC test with complete data

Consider a sample of *n* individuals who are arbitrarily related, with the pedigree information assumed to be known. (Note that individuals who are unrelated to anyone else in the sample are also allowed.) Let the kinship matrix derived from the pedigree information be given by

$$\Phi =\left(\begin{array}{cccc} 1+h_{1} & 2\phi_{1,2} & \ldots & 2\phi_{1,n}\\ 2\phi_{2,1} & 1+h_{2} & \ldots & 2\phi_{2,n}\\ \vdots & \ldots & \ldots & \vdots \\ 2\phi_{n,1} & 2\phi_{n,2} & \ldots & 1+h_{n} \end{array}\right),$$ (9)

where *ϕ*_*i*,*j*_ is the kinship coefficient between individuals *i* and *j*, and *h*_*i*_ is the inbreeding coefficient of individual *i*.

To model the binary trait variable ***Y***, we adopt a quasi-likelihood framework similar to that of CARAT, in which the mean structure is given by Eq (1). In the conditional variance of ***Y*** given ***X*** and ***G***, shown in Eq (2), we could use either the empirical genetic relationship matrix as in CARAT or the pedigree-based kinship matrix defined in Eq (9) as the genetic relationship matrix, **Φ**. As we will show in the next subsection, we use the pedigree-based kinship matrix as part of our approach to extract additional information for association from partially missing genotype data on related individuals, so it is convenient (although not necessary) to also use the pedigree-based kinship matrix for **Φ** in Eq (2). This would also eliminate the need for genome-wide data.

In the complete data case (i.e., when ***X***, ***G***, and ***Y*** are fully observed), to detect association between the binary phenotype and the genetic variant, we first obtain the quasi-score statistic defined as in Eq (5), but with the empirical genetic relationship matrix replaced by the kinship matrix calculated from the pedigree. Three commonly-used approaches for assessment of significance of an association test in genetic analysis can be described as (1) prospective, in which the conditional distribution of the phenotype given genotype and covariates is considered, (2) retrospective, in which the conditional distribution of the genotype given phenotype and covariates is considered, and (3) permutation-based. For CERAMIC, we take a retrospective approach similar to that of CARAT. One advantage of the retrospective approach is that it is robust to misspecification of the phenotype model, because correct type 1 error of CERAMIC relies only on the null conditional mean and variance of the vector of genotypes. Another advantage is that it allows for a natural way to incorporate information on individuals with missing genotypes, as described in the next subsection.

For the retrospective analysis, we make the following modeling assumptions about the distribution of ***G*** conditional on ***Y*** and ***X*** under the null hypothesis:

$${\text{E}}_{0}\left(G|Y,X\right)=X\alpha ,\;\text{and}$$ (10)

$${\text{Var}}_{0}\left(G|Y,X\right)=\sigma_{G}^{2}\Phi ,$$ (11)

where ***α*** is an unknown *k*-dimensional vector of coefficients, $\sigma_{G}^{2}>0$ is an unknown parameter, and **Φ** is the known kinship matrix defined in Eq (9). The null mean assumption in Eq (10) says that, under the null hypothesis of no association between genotype and phenotype, the genotype is permitted to be linearly related to the covariates, or it can be unrelated to the covariates. The possibility that ***G*** could be linearly related to the covariates is particularly relevant, for example, when ancestry vectors are used as covariates to account for population structure (e.g. M.P. Conomos, A.P. Reiner, M.S. McPeek and T.A. Thornton, under review). Then the null mean assumption allows, e.g., for different sub-populations to have different allele frequencies. The null variance assumption in Eq (11) is a version of the standard variance relationship that holds, for example, under Mendelian inheritance in a single population. Here, however, we do not require $\sigma_{G}^{2}=2p(1-p)$, where *p* is the allele frequency at the variant of interest, which would hold under Hardy-Weinberg equilibrium. Instead we use a more robust variance estimator [17] given by

$${\hat{\sigma }}_{G}^{2}=\frac{1}{n-k}G^{T}PG,$$ (12)

where ***P*** = **Φ**^−1^ − **Φ**^−1^ ***X***(***X***^*T*^ Φ^−1^ ***X***)^−1^ ***X***^*T*^ **Φ**^−1^. Note that ${\hat{\sigma }}_{G}^{2}$ is equivalent to the residual mean square error for the generalized linear regression of ***G*** on ***X***, with covariance matrix proportional to **Φ**. The CERAMIC test statistic in the complete data case is then given by

$${\text{CERAMIC}}_{c}=\frac{U_{0}^{2}}{{\hat{\text{Var}}}_{0}\left(U_{0}|Y,X\right)}=\frac{{\left(Z^{T}G\right)}^{2}}{{\hat{\sigma }}_{G}^{2}\;Z^{T}\Phi Z},$$ (13)

where ***Z*** is defined in Eq (7) and is referred to as the vector of transformed null phenotypic residuals. The subscript “*c*” on CERAMIC_*c*_ stands for “complete data.” Under suitable regularity conditions, significance of association could then be assessed by comparing CERAMIC_*c*_ to a $\chi_{1}^{2}$ random variable.

### Parameter estimation

In addition to testing for association, one can also obtain an estimate of the full parameter (*γ*, ***β***, *ξ*) by iteratively solving the system consisting of Eqs (3), (4) and (8). Let $\left(\hat{\gamma },\hat{\beta },\hat{\xi }\right)$ denote the estimator obtained as the solution of this system, and let $\hat{\Gamma }$ and $\hat{\Sigma }$ denote **Γ** and **Σ**, respectively, evaluated at $\left(\hat{\gamma },\hat{\beta },\hat{\xi }\right)$. Then the estimated asymptotic covariance matrix for $(\hat{\gamma },{\hat{\beta }}^{T}{)}^{T}$ is given by

$$\hat{\text{Cov}}\left({\left(\hat{\gamma },{\hat{\beta }}^{T}\right)}^{T}\right)={\left({\tilde{X}}^{T}{\hat{\Gamma }}^{1/2}{\hat{\Sigma }}^{-1}{\hat{\Gamma }}^{1/2}\tilde{X}\right)}^{-1},$$ (14)

where we define $\tilde{X}=(G,X)$. The approximate standard errors for the elements of $\left(\hat{\gamma },{\hat{\beta }}^{T}\right)$ are obtained as the square roots of the corresponding diagonal elements of the matrix in Eq (14). We note that the validity of Eq (14) as an estimator of the covariance relies on the validity of the modeling assumptions of Eqs (1) and (2) and on the sample size, *n*, being large. Calculation of the standard error for the estimator, $\hat{\xi }$, of the variance parameter would require higher-order (i.e., third and fourth) moment assumptions on ***Y***, so it is not available in our approach, in which we need only specify the mean and variance structure of the phenotype.

### CERAMIC test with partially missing data

First, we describe our notation with regard to missing data. Let *N* denote the full set of *n* sampled individuals. For a given genetic variant to be tested, let *R* ⊂ *N* denote the subset of individuals with non-missing genotype at that variant, and let *r* = |*R*| denote the number of such individuals. We define ***G***_*R*_ to be the *r* × 1 sub-vector of ***G*** that contains the genotypes for the individuals in set *R*, and we define **Φ**_*R*_ to be the *r* × *r* submatrix of **Φ** consisting of the rows and columns corresponding to the individuals in set *R*. We can partition the set *R* into two disjoint subsets, *U* and *V*, where *U* denotes the subset of individuals with non-missing genotype at the tested variant who also have complete phenotype and covariate data, and *V* denotes the subset of individuals with non-missing genotype at the tested variant who are missing either the phenotype or one or more covariates. We let *u* = |*U*| and *v* = |*V*|, and we have *R* = *U* ∪ *V*, *U* ∩ *V* = ∅ and *r* = *u* + *v*.

We let *S* denote the set consisting of the remaining *s* = *n* − *r* individuals not in the set *R*, i.e., *S* = *N* ∩ *R*^*c*^ denotes the subset of individuals who have missing genotype data at the variant of interest. There are certain categories of individuals who do not make a contribution to our association analysis, and we assume that these have already been deleted from the set *S* (and from *N*). To be retained in *S*, individuals with missing genotype are required to have non-missing phenotype and covariate information, and, in addition, to satisfy at least one of the following two conditions: (1) the individual has a genotyped relative; or (2) the individual is in the same pedigree with an individual with non-missing phenotype and covariates who either has non-missing genotype or has a relative with non-missing genotype at the tested variant. Let *W* = *U* ∪ *S*, so *W* is the set of *w* = *u* + *s* individuals remaining in *N* who have complete phenotype and covariate information. Notice that the sets *N*, *R*, *U*, *V*, *S*, and *W* can, in principle, vary across tested variants that have different patterns of genotypic missingness. This point is discussed in more detail in the subsection **Some computational considerations for CERAMIC**.

To form the CERAMIC test statistic in the case of partially missing data, we propose to use the genotype data for the individuals in the set *R* combined with the phenotype and covariate data for the individuals in the set *W*. As in the case of complete data, we first obtain an estimator of the phenotypic nuisance parameter, (***β***, *ξ*), under the null hypothesis, *H*_0_: *γ* = 0. This estimator, which we call $\left({\hat{\beta }}_{W0},{\hat{\xi }}_{W0}\right)$, is obtained by solving Eqs (3) and (4) with *γ* set to 0, where all vectors and matrices in Eqs (3) and (4) are restricted to contain only those individuals in set *W*. We then let ***Z***_*W*_ denote the vector of transformed null phenotypic residuals for the set *W*, where

$$Z_{W}={\hat{\Gamma }}_{W}^{1/2}{\hat{\Sigma }}_{W}^{-1}{\hat{\Gamma }}_{W}^{-1/2}\left(Y_{W}-{\hat{\mu }}_{W}\right),$$ (15)

where ${\hat{\Gamma }}_{W}$, ${\hat{\Sigma }}_{W}$, and ${\hat{\mu }}_{W}$ are **Γ**, **Σ**, and ***μ***, respectively, restricted to the individuals in set *W* and evaluated at $\left(\gamma ,\beta ,\xi )=(0,{\hat{\beta }}_{W0},{\hat{\xi }}_{W0}\right)$, and ***Y***_*W*_ is the vector ***Y*** restricted to the individuals in set *W*.

We define the CERAMIC statistic with partially missing data to be

$$\text{CERAMIC}=\frac{{\left(F^{T}G_{R}\right)}^{2}}{{\hat{\text{Var}}}_{0}\left(F^{T}G_{R}|Y,X\right)}=\frac{{\left(F^{T}G_{R}\right)}^{2}}{{\overset{ˇ}{\sigma }}_{G}^{2}F^{T}\Phi_{R}F}=\frac{{\left(Z_{W}^{T}\Phi_{RW}^{T}MG_{R}\right)}^{2}}{{\overset{ˇ}{\sigma }}_{G}^{2}Z_{W}^{T}\Phi_{RW}^{T}{M\Phi }_{R}{M\Phi }_{RW}Z_{W}},$$ (16)

where ***F*** = ***M*Φ**_*RW*_ ***Z***_*W*_ is a vector of length *r* that incorporates phenotype, covariate, and pedigree information, in which $M=\Phi_{R}^{-1}-\Phi_{R}^{-1}1_{r}(1_{r}^{T}\Phi_{R}^{-1}1_{r}{)}^{-1}1_{r}^{T}\Phi_{R}^{-1}$, where **1**_*r*_ is a vector of length *r* with every element equal to 1, and **Φ**_*RW*_ denotes the submatrix of **Φ** with rows corresponding to the individuals in *R* and columns corresponding to the individuals in *W*, i.e., the (*i*, *j*)th element of **Φ**_*RW*_ is 2*ϕ*_*ij*_, where *ϕ*_*ij*_ is the kinship coefficient between the *i*th individual in set *R* and the *j*th individual in set *W*. The variance estimator [17], ${\overset{ˇ}{\sigma }}_{G}^{2}$, is just the variance estimator, ${\hat{\sigma }}_{G}^{2}$, of Eq (15) restricted to the set, *Q*, of individuals in *N* who have non-missing genotypes and covariates but may or may not have observed phenotypes (*U* ⊂ *Q* ⊂ *R*), *q* = |*Q*|, i.e.

$${\overset{ˇ}{\sigma }}_{G}^{2}=\frac{1}{q-k}G_{Q}^{T}P_{Q}G_{Q},$$ (17)

where $P_{G}=\Phi_{Q}^{-1}-\Phi_{Q}^{-1}X_{Q}(X_{Q}^{T}\Phi_{Q}^{-1}X_{Q}{)}^{-1}X_{Q}^{T}\Phi_{Q}^{-1}$, and ***G***_*Q*_, ***X***_*Q*_, and **Φ**_*Q*_ are ***G***, ***X***, and **Φ**, respectively, restricted to the individuals in set *Q*. With complete data, i.e., *N* = *R* = *U* = *W* and *S* = *V* = ∅, the CERAMIC statistic of Eq (16) reduces to the CERAMIC_*c*_ statistic of Eq (13) (see S1 Text for details). Under assumptions described in the next subsection, the CERAMIC statistic follows an asymptotic $\chi_{1}^{2}$ distribution under the null hypothesis.

### Interpretation and justification for CERAMIC

One possible interpretation of the CERAMIC statistic in Eq (16) is that it uses best linear unbiased prediction to impute missing genotypes based on relatives’ genotypes, while downweighting predictions with low information level, correcting for imputation error and correcting for extra correlation due to imputation. This can be seen [5] by rewriting Eq (16) in terms of the best linear unbiased predictor (BLUP) of the missing genotypes for the individuals in set *S*. More generally, we can let

$${\hat{G}}_{W}=1_{w}\hat{p}+\Phi_{WR}\Phi_{R}^{-1}\left(G_{R}-1_{r}\hat{p}\right)=\left[1_{w}{\left(1_{r}\Phi_{R}^{-1}1_{r}\right)}^{-1}1_{r}^{T}\Phi_{R}^{-1}+\Phi_{WR}M\right]G_{R}$$ (18)

denote the BLUP of ***G***_*W*_, where ***G***_*W*_ is the vector ***G*** restricted to the individuals in set *W*, **1**_*w*_ is a vector of length *w* with every element equal to 1, and $\hat{p}=(1_{r}\Phi_{R}^{-1}1_{r}{)}^{-1}1_{r}^{T}\Phi_{R}^{-1}G_{R}$ is the best linear unbiased estimator [20] of the allele frequency, *p*, of the variant of interest. Consider ${\hat{G}}_{Wi}$, the *i*th element of ${\hat{G}}_{W}$. If individual *i* is in set *U*, then ${\hat{G}}_{Wi}$ can be shown [5] to be the observed genotype of individual *i*, while if individual *i* is in set *S*, then ${\hat{G}}_{Wi}$ is the BLUP of the unobserved genotype of individual *i*. In other words, if we reorder the individuals in set *W* so that the individuals in set *U* come first in the list and the individuals in set *S* follow, then we can write

$${\hat{G}}_{W}=\left(\begin{array}{c} G_{U}\\ 1_{s}\hat{p}+\Phi_{SR}\Phi_{R}^{-1}\left(G_{R}-1_{r}\hat{p}\right) \end{array}\right)=\left(\begin{array}{c} G_{U}\\ {\hat{G}}_{S} \end{array}\right),$$ (19)

where ***G***_*U*_ is the vector ***G*** restricted to the individuals in set *U*, and ${\hat{G}}_{S}$ is the BLUP for the missing genotypes of the individuals in set *S*. The CERAMIC statistic can then be rewritten (see S2 Text) in terms of the BLUP imputed genotypes, as

$$\text{CERAMIC}=\frac{{\left(Z_{W}^{T}{\hat{G}}_{W}\right)}^{2}}{{\hat{\text{Var}}}_{0}\left(Z_{W}^{T}{\hat{G}}_{W}|Y,X\right)}.$$ (20)

With retrospective modeling in which the conditional variance is assessed with respect to genotypes, the additional uncertainty and dependence due to genotype imputation is directly accounted for.

Alternatively, CERAMIC can be interpreted as a quasi-score test derived from a retrospective mean model [4, 17], though we do not detail this interpretation here. To obtain the asymptotic null distribution for CERAMIC, we slightly modify the null mean assumption in Eq (10) and assume $E_{0}({\hat{G}}_{W}|Y,X)=X_{W}\alpha$, where ***X***_*W*_ is the matrix ***X*** restricted to the individuals in set *W* and ***α*** is a *k*-dimensional vector of unknown coefficients. Then CERAMIC follows an asymptotic $\chi_{1}^{2}$ distribution under the null hypothesis under regularity conditions [21]. The accuracy, in finite samples, of the $\chi_{1}^{2}$ approximation to the null distribution is assessed in **Results**.

### Some computational considerations for CERAMIC

In order to carry out the iterative solution of the system of estimating equations given by Eqs (3) and (4) (or by Eqs (3), (4) and (8) when *γ* is to be estimated as well), we need to obtain the inverse of the *n* × *n* matrix **Σ** = *ξ***Φ** + (1 − *ξ*)***I*** for different values of *ξ*. We reduce the computational burden in two ways: (1) **Σ** is inverted block-wise where each block of **Σ** corresponds to a pedigree; and (2) a single spectral decomposition, **Φ** = ***AJA***^*T*^ (where ***A*** is an orthogonal matrix, and ***J*** is diagonal), is used to compute the inverse of **Σ** for different values of *ξ*, because **Σ**^−1^ = ***A***(*ξ**J*** + (1 − *ξ*)***I***)^−1^***A***^*T*^, where *ξ**J*** + (1 − *ξ*)***I*** is a diagonal matrix [22].

The calculation of the transformed null phenotypic residual vector, ***Z***_*W*_, depends on the set *W*, which is a function of the genotypic missingness pattern for the genetic variant being tested. In a GWAS, different SNPs often have different genotypic missingness patterns, so this could imply that in the worst case scenario, ***Z***_*W*_ would need to be computed separately for each SNP in the genome. One possible way to avoid this would be to compute ***Z***_*W*_ only once per genome scan based on all individuals with non-missing phenotype and covariate information (or based on some other fixed subset of individuals) and use the same ***Z***_*W*_ for association testing with respect to all SNPs across the genome. Another approach, which is the one we actually take, would be to estimate the variance component (VC) parameter, *ξ*, once per genome using all individuals with non-missing phenotype and covariate information, and then for each SNP, compute only the regression parameter, ***β***, by solving Eq (3), with *γ* = 0. In this way, we need only solve Eq (3) to obtain ***Z***_*W*_ separately for each SNP, which drastically reduces the computational burden.

### The MQLS-LOG and MQLS-LIN tests

In addition to CERAMIC, we also developed two other alternative approaches to association testing for binary traits in related individuals. These two methods, which we call MQLS-LOG and MQLS-LIN, both have the following features: (1) they incorporate covariates; (2) they are generalizations of the MQLS test [4]; (3) they are retrospective and handle missing data in the same way that CERAMIC does; and (4) they do not involve estimation of an additive polygenic component of variance. The main difference between them is that MQLS-LOG has a logistic mean structure while MQLS-LIN has a linear mean structure. In the remainder of this subsection, we give the details of these two tests, and in the **Results** section, we compare them, in terms of type 1 error and power, to CERAMIC and to three previously proposed tests, MASTOR, EMMAX, and GLOGS.

The MQLS-LOG and MQLS-LIN test statistics can each be constructed from the CERAMIC test statistic of Eqs (16) and (20) by replacing the transformed null phenotypic residual vector, ***Z***_*W*_, by some other type of residual vector that is a function of (***X***_*W*_, ***Y***_*W*_). To obtain the MQLS-LOG test statistic from the CERAMIC test statistic, we replace ***Z***_*W*_ by the vector of residuals from the logistic regression model, where this model is given by

$$Y_{i}|X_{W}\overset{}{∼}\text{Bernoulli}\left(p_{i}\right),\;\;\text{independently,}\;\text{for}\;i\in W\text{,}\;\text{with}\;\log\frac{p_{i}}{1-p_{i}}=X_{i}^{T}\beta .$$ (21)

Let $\tilde{\beta }$ be the maximum likelihood estimator for ***β*** under the model of Eq (21), and let ***ϵ*** be the resulting null phenotypic residual vector, defined to have *i*th element $\epsilon_{i}=Y_{i}-{\hat{p}}_{i}$, for *i* ∈ *W*, where ${\hat{p}}_{i}$ is given by $\text{log}\frac{{\hat{p}}_{i}}{1-{\hat{p}}_{i}}=X_{i}^{T}\tilde{\beta }$. Then the MQLS-LOG test statistic is given by

$$\text{MQLS-LOG}=\frac{{\left(\epsilon^{T}{\hat{G}}_{W}\right)}^{2}}{{\hat{\text{Var}}}_{0}\left(\epsilon^{T}{\hat{G}}_{W}|Y,X\right)}=\frac{{\left(\epsilon^{T}\Phi_{RW}^{T}{MG}_{R}\right)}^{2}}{{\overset{ˇ}{\sigma }}_{G}^{2}\epsilon^{T}\Phi_{RW}^{T}{M\Phi }_{R}{M\Phi }_{RW}\epsilon },$$ (22)

where ${\hat{G}}_{W}$ is defined in Eq (18), and where we have used the fact that ***ϵ***^*T*^**1**_*w*_ = 0 for logistic regression.

To obtain the MQLS-LIN test statistic from the CERAMIC test statistic, we replace ***Z***_*W*_ by the vector of residuals from the ordinary linear regression model, where this model is given by

$$\text{E}\left(Y_{W}|X_{W}\right)=X_{W}^{T}\beta \;\text{and}\;\text{Var}\left(Y_{W}|X_{W}\right)=\sigma^{2}I.$$ (23)

Let $\overset{ˇ}{\beta }=(X_{W}^{T}X_{W}{)}^{-1}X_{W}^{T}Y_{W}$ denote the ordinary least squares estimator for ***β*** under model (23), and let $e=Y_{W}-X_{W}\overset{ˇ}{\beta }$ be the resulting null phenotypic residual vector. Then the MQLS-LIN test statistic is given by

$$\text{MQLS-LIN}=\frac{{\left(e^{T}{\hat{G}}_{W}\right)}^{2}}{{\hat{\text{Var}}}_{0}\left(e^{T}{\hat{G}}_{W}|Y,X\right)}=\frac{{\left(e^{T}\Phi_{RW}^{T}MG_{R}\right)}^{2}}{{\overset{ˇ}{\sigma }}_{G}^{2}e^{T}\Phi_{RW}^{T}M\Phi_{R}M\Phi_{RW}e}.$$ (24)

Under the same assumptions as for CERAMIC, the MQLS-LOG and MQLS-LIN test statistics both have $\chi_{1}^{2}$ asymptotic null distributions.

### Test statistics considered in simulations

In simulations, we assess the type 1 error and power of the three methods we propose, CERAMIC, MQLS-LOG, and MQLS-LIN, and we compare them to five previously-proposed methods, EMMAX [8], GEMMA [10], MASTOR [17], GMMAT [15] and CARAT [16]. Table 1 summarizes some of the major features of the methods that are particularly relevant to the type 1 error and power studies. In the simulations, we provide CERAMIC, GMMAT and GEMMA with the pedigree-based kinship matrix, while for CARAT and EMMAX, we use an empirical kinship matrix based on 10,000 independently simulated SNPs with their MAFs randomly drawn from the uniform distribution on the interval between 0.05 and 0.45.

**Table 1:** Some Relevant Features of the Methods Compared in Simulations.

Method	Mean Model	Additive Polygenic VCs?	Analysis Type	Sophisticated Use of Missing Data?
CERAMIC	logistic	yes	retrospective	yes
GMMAT	logistic	yes	prospective	no
CARAT	logistic	yes	retrospective	no
MQLS-LOG	logistic	no	retrospective	yes
EMMAX, GEMMA	linear	yes	prospective	no
MASTOR	linear	yes	retrospective	yes
MQLS-LIN	linear	no	retrospective	yes

“Sophisticated Use of Missing Data” refers to methods that do something more sophisticated than plugging in the mean genotype value for missing genotype values or removing missing values.

### Simulation study design: Covariate model

**Fig 1:**
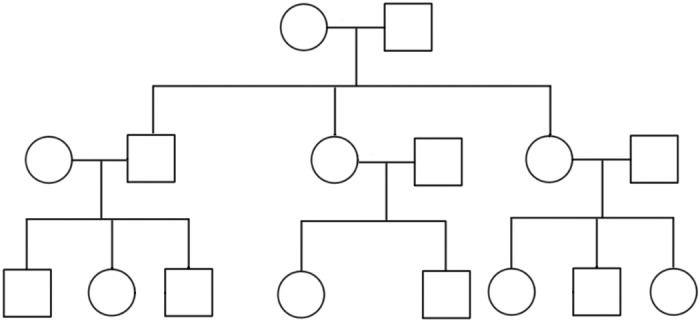
Three-Generation Pedigree Used in Simulations. In the simulations, each sampled family is assumed to have a 3-generation, 16-person pedigree of this form.

We simulate genotype, covariate and phenotype data for a sample that includes some unrelated individuals and some individuals in three-generation pedigrees (see Fig 1). For each individual, four covariates are simulated: age, sex, height, and an i.i.d. normal covariate. The individuals in pedigrees are each assigned to one of three generations based on their position in the pedigree: first (i.e., grandparent) generation, second (i.e., parent) generation, and third (i.e., offspring) generation. Among the sampled unrelated individuals, 50% are randomly assigned to the first generation, 25% to the second generation, and 25% to the third generation. The age of an individual is generated according to the generation the individual belongs to. For an individual in the first generation, the age is simulated according to a uniform distribution on the set of integers from 78 to 88, i.e., uniform on {78, 79, ⋯, 88}. An individual in the second generation has his or her age generated from a uniform distribution on the set of integers {48, 49, ⋯, 58}, and for an individual in the third generation, we use a uniform distribution on the set {18, 19, ⋯, 28}. Ages for different individuals are generated independently, regardless of their familial relationships. Let ***X***_(2)_ denote the column vector of age values for a simulated sample. Sampled individuals from three-generation pedigrees have their sex pattern fixed as shown in Fig 1. Among the unrelated individuals, half are randomly assigned to be males and half females. Let ***X***_(3)_ denote the column vector of sex values for a simulated sample. Height is simulated as a heritable trait that exhibits correlation among family members and depends on age and sex. Let ***X***_(4)_ denote the column vector of height values for a simulated sample. The model for height is multivariate normal, given by

$$X_{\left(4\right)}|X_{\left(2\right)},X_{\left(3\right)}∼\text{MVN}\left(\nu \left(X_{\left(2\right)},X_{\left(3\right)}\right),\sigma_{h_{a}}^{2}\Phi +\sigma_{h_{e}}^{2}I\right),$$ (25)

where $\sigma_{h_{a}}^{2}=36$ represents additive polygenic variance for an outbred individual, and $\sigma_{h_{e}}^{2}=13$ represents i.i.d. error variance, resulting in narrow-sense heritability ≈ 73%. The mean height vector, ***ν***(***X***_(2)_, ***X***_(3)_), has entry 172.5 for a male with age ≥ 65 and entry 176.5 for a male with age < 65. For a female with age ≥ 65, the entry in the mean height vector, ***ν***(***X***_(2)_, ***X***_(3)_), is 160.2, while for a female with age < 65, the mean height is set to be 163.2. Let *X*_(5)_ denote the column vector of the values of the i.i.d. normal covariate for a simulated sample. The entries of ***X***_(5)_ are generated from the *N*(8, 9) distribution, independently of all other covariates. Let ***X*** = (**1**, ***X***_(2)_, ⋯, ***X***_(5)_) be the covariate matrix consisting of an intercept and the four covariates described above.

### Simulation study design: Trait models

Two types of trait model are considered in our simulation studies. One is a mixed-effects logistic regression model, which has the following form:

$$Y_{i}|G,X,u\overset{}{∼}\text{Bernoulli}\left(p_{i}\right),\;\text{independently,}\;\text{with}\;p_{i}=h\left(f\left(G_{i}\right)+X_{i}^{T}\beta +u_{i}\right),$$ (26)

for *i* = 1, …, *n*, where $X_{i}^{T}$ is the *i*th row of ***X***, ***u*** = (*u*_1_, ⋯, *u*_*n*_)^*T*^ is a vector of additive polygenic effects, ***G***_*i*_ = (*G*_*i*1_,*G*_*i*2_) represents the genotypes at causal SNPs 1 and 2 for individual *i*, and *f*(***G***_*i*_) is a function of ***G***_*i*_ that represents the combined genetic effect, on the phenotype, of causal SNPs 1 and 2. The coefficient vector ***β*** is chosen to satisfy two conditions: (1) the mean of the covariate effects on the logit scale, $E(X_{i}^{T}\beta )=0$, and (2) the variance of the covariate effects on the logit scale, $Var(X_{i}^{T}\beta )$, achieves a specified level that depends on the simulation setting. The additive polygenic effects, ***u***, independent of covariates, have a multivariate normal distribution with mean **0** and covariance matrix $\sigma_{a}^{2}\Phi$, where $\sigma_{a}^{2}$ varies across simulation settings. Let $\theta_{a}=Var(u_{i})/Var(X_{i}^{T}\beta +u_{i})=\sigma_{a}^{2}/(Var(X_{i}^{T}\beta )+\sigma_{a}^{2})$ denote the fraction of $Var(X_{i}^{T}\beta +u_{i})$ that is due to additive polygenic effects, and let *θ*_*c*_ = 1 − *θ*_*a*_ denote the fraction due to covariate effects. We fix $Var(X_{i}^{T}\beta )+\sigma_{a}^{2}=100$ and let $\sigma_{a}^{2}$ take possible values 0, 20, 40, 60, 80, and 100, so that (*θ*_*a*_, *θ*_*c*_) takes possible values (0, 1), (.2, .8), (.4, .6), (.6, .4), (.8, .2) and (0, 1), representing a range of the relative importance of additive genetic effects vs. covariate effects. Note that the model also results in Bernoulli error in the phenotype that is conditionally independent across individuals and that accounts for ∼ 20% of total phenotypic variance in our simulation scenarios (where this value is obtained by simulation).

Causal SNPs 1 and 2, whose genotypes for individual *i* are encoded in ***G***_*i*_, are unlinked with minor allele frequencies (MAFs) .1 and .2 respectively, and they are generated independently of the covariates and additive polygenic effects. They act on the phenotype epistatically: an individual with at least one copy of the minor allele at causal SNP 1 and at least one copy of the minor allele at causal SNP 2 has mean penetrance *E*(*Y*_*i*_|***G***) ≈ .15; an individual with a genotype not satisfying that condition has mean penetrance *E*(*Y*_*i*_|***G***) ≈ .05. (Note that a target mean penetrance can be achieved by setting *f*(***G***_*i*_) to be an appropriate value, obtained in a simulation-based approach.) The resulting prevalence is *E*(*Y*_*i*_) ≈ .057.

The other type of model we consider is a liability threshold model,

$$Y_{i}=\left\{\begin{array}{cc} 1 & \text{if}\;L_{i}\geq \lambda \left(G_{i}\right)\\ 0 & \text{if}\;L_{i}<\lambda \left(G_{i}\right) \end{array}\right.,\;\text{with}\;L_{i}=X_{i}^{T}\beta +u_{i}+\epsilon_{i},$$ (27)

where *L*_*i*_ is the underlying liability for individual *i*, and *λ*(***G***_*i*_) represents the individual’s liability threshold, beyond which the disease is activated, as a function of individual *i*’s genotypes at causal SNPs 1 and 2. The liability *L*_*i*_ consists of three components: $X_{i}^{T}\beta$, the covariate effects, *u*_*i*_, the random additive polygenic effects, and *ϵ*_*i*_, which represents measurement error or environmental effects assumed to be acting independently across individuals. The ***X***_*i*_’s and the *u*_*i*_’s have the same distributions as described above. The error terms *ϵ*_1_,⋯,*ϵ*_*n*_ are i.i.d. $N(0,\sigma_{e}^{2})$ and are independent of (***X***, ***u***). We fix the total liability variance $Var(L_{i})=Var(X_{i}^{T}\beta )+\sigma_{a}^{2}+\sigma_{e}^{2}$ to be 100, and the error variance $\sigma_{e}^{2}$ to be 20, so that the liability variance due to additive polygenic effects and covariates, $Var(X_{i}^{T}\beta )+\sigma_{a}^{2}=80$. Let $\pi_{a}=Var(u_{i})/Var(L_{i})=\sigma_{a}^{2}/(Var(X_{i}^{T}\beta )+\sigma_{a}^{2}+\sigma_{e}^{2})$ represent the fraction of total liability variance due to additive polygenic effects, and let $\pi_{c}=Var(X_{i}^{T}\beta )/(Var(X_{i}^{T}\beta )+\sigma_{a}^{2}+\sigma_{e}^{2})=.8-\pi_{a}$ represent the fraction due to covariate effects, while the fraction due to the independent error is fixed at.2. We choose different values for ***β*** and $\sigma_{a}^{2}$ to allow (*π*_*a*_, *π*_*c*_) to take on the possible values (0,.8), (.2,.6), (.4,.4), (.6,.2), and (.8, 0), representing a range of the relative importance of additive genetic vs. covariate effects. ***G***_*i*_, individual *i*’s genotypes at causal SNPs 1 and 2, has the same distribution as for the mixed-effects logistic regression model, and in each setting, the values of *λ*(***G***_*i*_) are chosen so that an individual with at least one copy of the minor allele at causal SNP 1 and at least one copy of the minor allele at causal SNP 2 has mean penetrance *E*(*Y*_*i*_|***G***_*i*_) ≈ .15, while an individual with a genotype not satisfying that condition has mean penetrance ≈ .05. The resulting prevalence is again ≈ .057.

In addition, we consider two variations on the liability threshold model. In the first variation, we model the effects of shared environment by incorporating a sibship random effect that accounts for 10% of the total liability variance. In that case, we set the error variance to also account for 10% of the total liability variance, and the additive polygenic and covariate effects are as described in the previous paragraph. In the second variation on the liability threshold model, we modify the threshold values, *λ*(***G***_*i*_), so that the prevalence is reduced to.01.

### Simulation study design: Ascertainment and missingness

In simulations, we consider both the case when there is complete genotype, phenotype and covariate data as well as cases with missing data. In the complete data case, we simulate genotype, covariate and phenotype data, according to one of the binary trait models described above, for either a 600-person sample (consisting of 30 families, with each family having the 16-person pedigree shown in Fig 1, and an additional 120 unrelated individuals) or a 1000-person sample (consisting of 50 families and an additional 200 unrelated individuals). In some scenarios, families are ascertained conditional on containing at least four affected individuals, while unrelated individuals are sampled at random from the population (call this ascertainment setting A), while in other scenarios, families are ascertained as in setting A while unrelated individuals are sampled in a 1:1 case-control ratio (call this ascertainment setting B). All sampled individuals are assumed to have non-missing covariates, phenotypes and genotypes.

For scenarios with missing data, we simulate genotype, covariate and phenotype data for either a 1,200-person sample (consisting of 60 families, with each family having the 16-person pedigree shown in Fig 1, and an additional 240 unrelated individuals), or a 2,000-person sample (consisting of 100 families and an additional 400 unrelated individuals). In addition, for the run time assessments, we also simulate samples of size 10^3^, 4 × 10^3^, 6 × 10^3^, 8 × 10^3^ and 10^4^, each consisting of 80% related and 20% unrelated individuals. We use ascertainment settings A and B as described above. For each individual in a family, his or her phenotype and covariates are assumed to be all non-missing with probability .8 (and are assumed to be all missing with probability .2), independently across individuals and families, and his or her genotype at the tested locus is assumed to be non-missing if and only if at least one of the following two conditions holds: (1) the individual has non-missing phenotype and is affected, or (2) at least half of the individual’s first degree relatives who have non-missing phenotypes are affected. Among the unrelated individuals, all their phenotypes and covariates are assumed to be missing, and all their genotypes are assumed to be non-missing, i.e., they are included as controls of unknown phenotype.

### Application to T2D data from the FHS

The FHS is a multicohort, longitudinal study whose primary objective is to identify the risk factors and characteristics responsible for cardiovascular disease. The goal of our data analysis is to identify SNPs that are associated with T2D. Our use of the FHS data was approved by the Institutional Review Board of the Biological Sciences Division of the University of Chicago. The FHS sample consists of unrelated individuals as well as individuals from multigeneration pedigrees. For cohort 1 (i.e., original cohort), we use phenotype and covariate information from 27 clinical exams, for cohort 2 (i.e., offspring cohort), we use information from 7 clinical exams, and for cohort 3 (i.e., generation three cohort) we use information from 1 clinical exam. We determine the T2D phenotype status in a similar way to that in a previous work [23]. For individuals in cohort 1, we use data from exams 1–27 to label their T2D status as follows: individuals who have at least one exam with nonfasting blood glucose (BG) level ≥ 200mg/dl or who were under treatment for diabetes, where the measurement or treatment occurred between the ages of 35 and 75 years, are classified as “affected.” For individuals who have all exams with nonfasting BG<200 mg/dl and have never taken any treatment by the time of the last exam, a phenotype label “unaffected” is given if the individual has age ≥ 70 years at the time of the last exam, while the “unknown” label is given otherwise. We use data from exams 1–7 and exam 1 to determine the phenotype status for individuals in cohorts 2 and 3, respectively. Phenotype is coded as follows for both cohorts: individuals who have at least one exam with fasting plasma glucose (FPG) level ≥ 126mg/dl or who were under treatment for diabetes, where the measurement or treatment occurred between the ages of 35 and 75 years, are classified as “affected.” For individuals who have all exams with FPG<126 mg/dl and have never taken any treatment by the time of the last exam, a phenotype label “unaffected” is given if the individual has age ≥ 70 years at the time of the last exam while “unknown” is given otherwise. Sex and body mass index (BMI) are included in our analysis as covariates, where we use the mean of an individual’s available BMI values from all clinical exams that the individual participated in. Note that onset age is not included as a covariate in our analysis, because it is not well-defined for an unaffected individual. In addition, the age of an individual at the time of the last exam and cohort ID are artificially correlated to the phenotype status in the restricted sample with known phenotypes, and therefore neither should be added as a covariate.

Among the 9240 study individuals for whom Affymetrix 500K genotype data are available, we exclude individuals who have either (1) completeness (the proportion of markers with successful genotype calls) ≤ 96%, or (2) empirical self-kinship coefficient ${\hat{\Phi }}_{ii}\geq 1.05$. In addition, we exclude 298 individuals whose off-diagonal empirical kinship coefficient values are not consistent with the given pedigree information. Of the 8080 individuals retained in the analysis, 639 are not related to anyone else in the data set with the remaining 7441 related through 840 pedigrees. 6042 individuals have either missing phenotype or missing covariate information, 625 are affected with non-missing covariates, and 1413 are unaffected with non-missing covariates. We exclude from our analysis SNPs that have (1) call rate ≤ 96%, or (2) Mendelian error rate > 2%, or (3) MAF < 1%, which results in a total of 368,802 SNPs retained in the analysis. Furthermore, following Wu and McPeek (submitted), we note that individuals in the original cohort appear to have on average lower genotype quality (lower completeness and higher empirical self-kinship values) than those in the other two cohorts. To prevent spurious association potentially caused by poor genotype quality in cohort 1, for each SNP, we test for an allele frequency difference between cohort 1 and the other cohorts combined. If the allele frequency difference is significant at level 10^−7^, the SNP is removed from our study. Under this screening procedure, we exclude an additional 1,032 SNPs, resulting in a final set of 367,770 SNPs to include in the analysis.

## Results

### Assessment of type 1 error when relevant covariates are included in the trait model

To assess the type 1 error of CERAMIC, MQLS-LOG and MQLS-LIN, we perform simulations as described in **Methods**. In each simulation scenario, phenotypes are generated according to either the mixed effects logistic regression model of Eq (26) with (*θ*_*a*_, *θ*_*c*_) = (.6, .4) (referred to in Table 2 as “Logistic”) or the liability threshold model of Eq (27) with (*π*_*a*_, *π*_*c*_) = (.4, .4) (referred to in Table 2 as “Liability”). Association is tested with a SNP that is neither linked nor associated with the trait, with MAF set to be either .1 or .2. In every scenario, we simulate 1,200 individuals with missing data using ascertainment setting A as described in **Methods**. In each simulation scenario, we consider one of two approaches to analyzing the data, either (1) individuals with partially missing data are included in the analysis for every statistic (denoted by “All” in Table 2), or else (2) individuals with partially missing data are dropped from the analysis for every statistic (denoted by “MX” for “missing excluded” in Table 2). Table 2 shows that in every case, the empirical type 1 error is not significantly different from the nominal, verifying the correct type 1 error of CERAMIC, MQLS-LOG and MQLS-LIN in these scenarios.

**Table 2:** Empirical Type 1 Error of CERAMIC, MQLS-LOG and MQLS-LIN, Based on 25,000 Simulated Replicates.

				Empirical Type I Error of		
Trait Model	Subset Used	MAF	Nominal Level	CERAMIC	MQLS-LOG	MQLS-LIN
Logistic	All	0.2	.05	.049	.050	.049
Logistic	All	0.1	.05	.051	.050	.050
Logistic	MX	0.2	.05	.049	.049	.049
Logistic	MX	0.1	.05	.051	.051	.050
Logistic	All	0.2	.001	.0011	.0010	.0011
Logistic	All	0.1	.001	.0007	.0008	.0007
Logistic	MX	0.2	.001	.0010	.0010	.0010
Logistic	MX	0.1	.001	.0009	.0009	.001
Liability	All	0.2	.05	.051	.050	.050
Liability	All	0.1	.05	.048	.0474	.048
Liability	MX	0.2	.05	.052	.052	.051
Liability	MX	0.1	.05	.050	.050	.051
Liability	All	0.2	.001	.0011	.0011	.0010
Liability	All	0.1	.001	.0011	.0011	.0012
Liability	MX	0.2	.001	.0008	.0008	.0008
Liability	MX	0.1	.001	.0009	.0009	.0007

“Logistic” denotes the mixed-effects logistic regression model of Eq 26 with (*θ*_*a*_, *θ*_*c*_) = (.6, .4). “Liability” denotes the liability threshold model of Eq 27 with (*π*_*a*_, *π*_*c*_) = (.4, .4). “All” indicates that all individuals, including those with partially missing data are included in the analyses for all three statistics. “MX” (for “missing excluded”) indicates that only individuals with complete data are included in the analyses for all three statistics. “MAF” denotes the minor allele frequency of the tested SNP. The radius of the 95% confidence interval for nominal level .05 is .0027, and that for nominal level .001 is .0004.

S1 and S2 Tables contain additional type 1 error results for scenarios that include effects of shared environment on the trait and more stringent ascertainment on the unrelated individuals in a sample (ascertainment setting B) or more stringent ascertainment due to reduced prevalence (value .01). The number of individuals in a sample ranges from 600 to 2,000, depending on the scenario. In every scenario, the type 1 error remains correct for CERAMIC, MQLS-LOG and MQLS-LIN.

We perform additional simulations in which we compare the type 1 error rate of GEMMA to those of CERAMIC, MQLS-LOG and MQLS-LIN. Phenotypes are generated according to the liability threshold model of Eq (27) with various settings of (*π*_*a*_, *π*_*c*_). In every scenario, we simulate 1,200 individuals with missing data and use ascertainment setting A. Association is tested with an unlinked, unassociated SNP with MAF .2. S3 Table shows that in every scenario, the type 1 error of GEMMA is significantly inflated when the mean genotype value is imputed for the missing genotypes, and it is significantly deflated when the missing genotypes are removed. In contrast, the type 1 error of CERAMIC, MQLS-LOG and MQLS-LIN is correct in all scenarios. Because we observe uncontrolled type 1 error for GEMMA in these settings, we do not consider GEMMA further in our simulations.

### Type 1 error with trait model misspecification

In an association study, the correct trait model is generally unknown. In particular, it may not be known which covariates should be included in the model. In Table 3, we compare the type 1 error of CERAMIC, MQLS-LOG and GMMAT in the situation in which the relevant covariates are inadvertantly left out of the fitted model. The results show that in almost every scenario, the type 1 error of GMMAT is compromised (either significantly inflated or significantly deflated) when the trait model is misspecified. In contrast, CERAMIC and MQLS-LOG retain correct type 1 error when the trait model is misspecified. This likely reflects the fact that retrospective methods tend to be much more robust to phenotype model misspecification than prospective methods are.

**Table 3:** Type 1 Error when the Trait Model Is Misspecified.

			Empirical Type I Error of		
Partially Missing Data?	Model	Setting	MQLS-LOG	CERAMIC	GMMAT
Yes	Threshold (60,20)	A	.050	.050	**.061**
Yes	Threshold (40,40)	A	.048	.048	**.066**
Yes	Threshold (20,60)	A	.050	.050	**.057**
Yes	Threshold (0,80)	A	.049	.049	**.057**
Yes	Threshold (20,60)	B	.051	.051	**.057**
Yes	Threshold (20,60)	.01	.051	.051	**.060**
Yes	Logistic (80,20)	A	.051	.052	**.058**
Yes	Logistic (60,40)	A	.050	.050	**.059**
Yes	Logistic (40,60)	A	.050	.050	**.063**
Yes	Logistic (20,80)	A	.048	.048	**.058**
Yes	Logistic (0,100)	A	.052	.052	.052
No	Threshold (60,20)	A	.048	.048	**.047**
No	Threshold (40,40)	A	.051	.051	.048
No	Threshold (20,60)	A	.051	.051	**.040**
No	Threshold (0,80)	A	.051	.051	**.029**
No	Threshold (20,60)	B	.050	.050	**.044**
No	Threshold (20,60)	.01	.049	.049	**.039**
No	Logistic (80,20)	A	.049	.049	.050
No	Logistic (60,40)	A	.051	.051	.050
No	Logistic (40,60)	A	.050	.050	**.045**
No	Logistic (20,80)	A	.048	.048	**.037**
No	Logistic (0,100)	A	.049	.049	**.029**

“Trait Model is Misspecified” refers to the fact that the relevant covariates are left out of the fitted model. Under “Model”, for example, “Threshold (60, 20)” refers to the mixed effects liability threshold trait model with (*π*_*a*_, *π*_*c*_) = (60, 20) and “Logistic (80,20)” refers to the mixed effects logistic trait model with (*θ*_*a*_, *θ*_*c*_) = (80, 20). Under “Setting”, “A” refers to ascertainment setting A, “B” refers to the setting in which a shared environment random effect is included in the trait model and ascertainment setting B is used, and “.01” refers to the setting in which the prevalence is set to.01 and ascertainment setting A is used. In all scenarios in which partially missing data are included, the number of individuals sampled in each simulated replicate is 1,200, while in all complete data settings, the number of individuals sampled in each simulated replicate is 600. Empirical type 1 error is assessed based on 25,000 simulations. The radius of the 95% confidence interval for nominal level .05 is .0027. Bold indicates a type 1 error rate that is outside the 95% confidence interval.

### Power comparison with partially missing data when relevant covariates are included in the trait model

**Fig 2:**
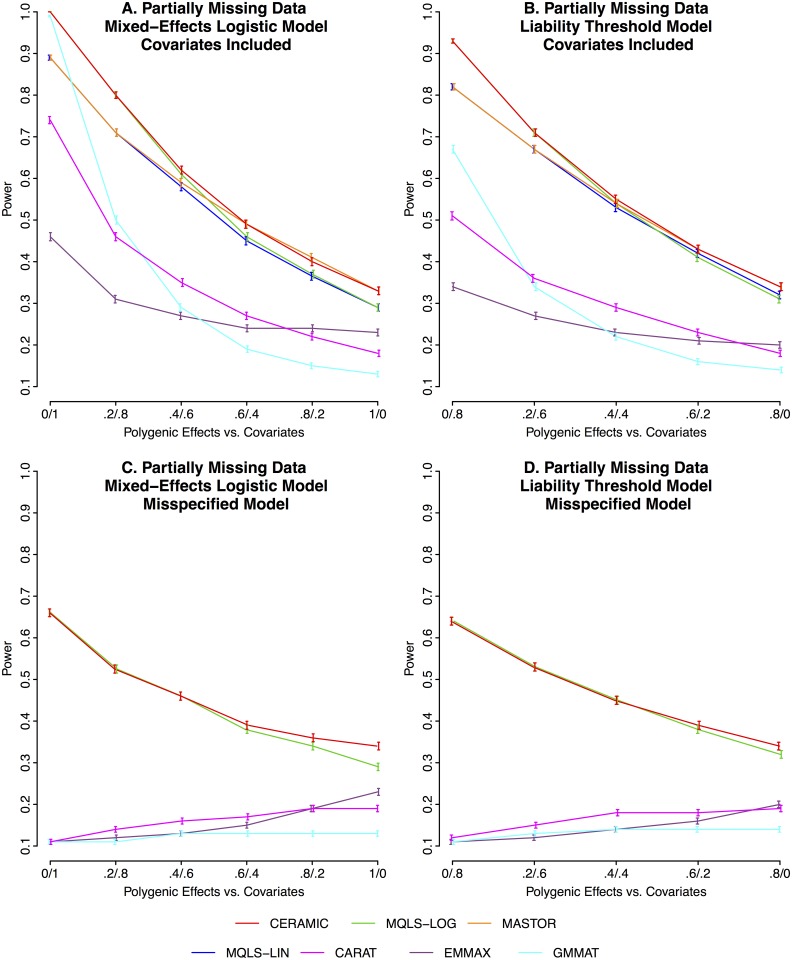
Empirical Power of CERAMIC and Other Methods. Empirical power is based on 10,000 replicates. The error bars indicate 95% confidence intervals. Panels A and B are for the case in which all relevant covariates are included in the fitted model. Panels C and D are for the case in which the relevant covariates are not included in the fitted model. In Panels A and C, the trait is simulated by the mixed-effects logistic regression model, and it Panels B and D, it is by the liability threshold model. The horizontal scale in the plots indicates the relative impact of covariates versus additive polygenic effects on the phenotype, with the far left corresponding to no polygenic effects and strong effects of covariates and the far right corresponding to no effect of covariates and strong polygenic effects. In all cases, partially missing data are simulated and ascertainment setting A is used. In the settings of panels C and D, the MQLS-LOG and MQLS-LIN methods give identical results, so only the MQLS-LOG results are depicted, and similarly, the CERAMIC and MASTOR methods give identical results, so only the CERAMIC results are depicted.

We compare the power of CERAMIC to that of MQLS-LOG, MQLS-LIN, MASTOR, EMMAX, GMMAT and CARAT in various simulated scenarios with missing data and ascertainment, as described in **Methods**. GMMAT offers two options to deal with missing genotype data: the missing genotypes can either be removed or replaced by the estimated mean genotype value. In practice, we found that the two options give identical results, so the simulation results we report for GMMAT apply to both options. Panel A in Fig 2 gives the empirical power results for various settings of the mixed effects logistic regression model with missing data, while Panel B in Fig 2 gives the results for the liability threshold model with missing data. In both cases, ascertainment setting A is used. Numerical results for these power simulations can be found in S4 and S6 Tables. S12 and S13 Tables give power results for additional missing data scenarios that include effects of shared environment on the trait and more stringent ascertainment on the unrelated individuals in a sample (ascertainment setting B) or more stringent ascertainment due to reduced prevalence (value .01).

From Panels A and B in Fig 2, it is clear that in every scenario, in terms of power, CERAMIC either outperforms, or has equivalent performance to, the best of the other methods, regardless of the relative strength of covariates and additive polygenic effects on the trait. In particular, CERAMIC has dramatically higher power than the previously-proposed binary trait methods GMMAT and CARAT. This result also holds in the partially missing data scenarios in S12 and S13 Tables.

In Panels A and B of Fig 2, a major feature distinguishing the power of the methods is that those methods that make sophisticated use of missing data (CERAMIC, MQLS-LOG, MASTOR and MQLS-LIN) substantially outperform those that do not (CARAT, EMMAX and GMMAT). Within each of these two groups, when covariate effects are large relative to additive polygenic effects (*θ*_*a*_ ≤ .4 or *π*_*a*_ ≤ .2), the methods that fit a logistic mean structure outperform the methods that fit a linear mean structure, i.e., CERAMIC and MQLS-LOG outperform MASTOR and MQLS-LIN, while CARAT and GMMAT outperfom EMMAX. For the mixed-effects logistic regression trait model, this is not surprising, because the simulated model also has a logistic mean structure. However, it is notable that, among methods that treat missing data in the same way, the methods that fit a logistic mean structure also outperform those that fit a linear structure in the case of the liability threshold model, in which the simulated model does not have a logistic mean structure. This improvement may be due to the flexibility afforded by the nonlinearity of a logistic mean structure. Through the power comparison of CERAMIC to MQLS-LOG and that of MASTOR to MQLS-LIN, we can see that fitting the additive VC (as in MASTOR and CERAMIC) does not harm power in any scenario, and it improves power when the additive polygenic effects are large relative to covariate effects (*θ*_*a*_ ≥ .6, or *π*_*a*_ ≥ .8). When additive polygenic effects are large, GMMAT has the lowest power of all methods. This might reflect known limitations [24] of the penalized quasi-likelihood approach, which is used by GMMAT.

### Extent to which missing genotype information is recovered by the binary trait methods

**Fig 3:**
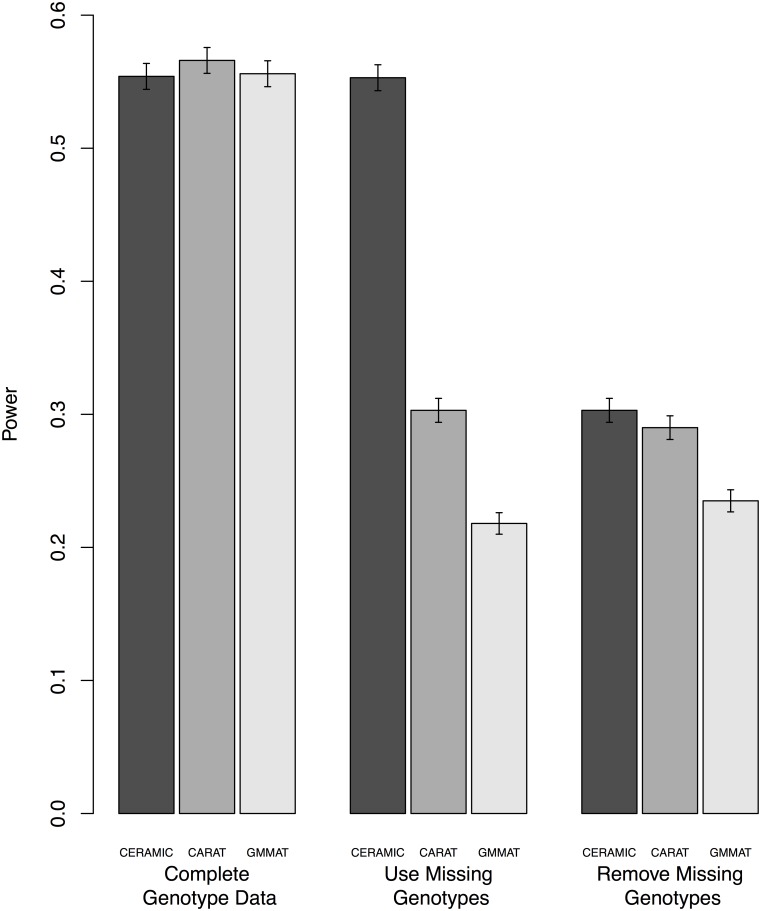
Comparison of Extent of Power Recovery with Missing Genotypes for CERAMIC, CARAT and GMMAT. Empirical power is based on 10,000 replicates. The error bars indicate 95% confidence intervals. The trait is simulated according to the liability threshold trait model with (*π_a_*, *π_b_*) = (40; 40) with 1,200 individuals in each simulated replicate, under acertainment setting A with missing data. In the “Remove Missing Genotypes” setting, individuals with missing genotypes are removed from the input files before the methods are run. In the “Use Missing Genotypes” setting, individuals with missing genotypes remain in the input files, and GMMAT is run with the option to impute the mean genotype value for the missing genotypes, CERAMIC is run with default settings, and CARAT is run with the mean genotype value plugged in for the missing genotypes in the input file. In the “Complete Genotype Data” setting, the missing genotype values are “unmasked” and included in the input files for all methods.

We perform additional simulations to compare the ability of the three binary trait methods, CERAMIC, GMMAT and CARAT, to recover power from partially missing genotype information when mean values are plugged in for missing genotype values in both GMMAT and CARAT. We simulate under the liability threshold trait model with (*π*_*a*_, *π*_*c*_) = (40, 40) with 1,200 individuals in each simulated replicate, under acertainment setting A with missing data. Because the data are simulated, the missing genotype values are actually available, so we can determine what the power would have been for each of the three methods had the genotype data not been missing. This power is represented in the leftmost set of three bars in Fig 3, labeled “Complete Genotype Data.” This is compared to the power when the individuals with missing genotype data are removed from the input files before the methods are run, and the power when individuals with missing genotype data remain in the input files and CERAMIC is run in the usual way, while GMMAT and CARAT are run with the mean genotype value plugged in for the missing genotypes.

From Fig 3, we can see that in this setting, CERAMIC is able to recover virtually all of the power of the complete genotype data by using the information from the partially missing genotype data. In contrast, the strategy of imputing the mean genotype value for missing genotype data in GMMAT or CARAT results in power that is not significantly different from that obtained by throwing those individuals out of the analysis. This demonstrates that appropriate handling of missing data can result in a substantial power advantage compared to a simple strategy such as imputing the mean genotype value or discarding missing values.

### Power comparison with partially missing data and model misspecification

Because the trait model typically cannot be known with certainty, it is always possible that relevant covariates may be left out of the fitted model. Panels C and D of Fig 2 show results from the same simulated scenarios as those of Panels A and B, respectively, but for the situations in which the fitted model excludes the relevant covariates. Numerical results for these power simulations can be found in S5 and S7 Tables. In Fig 2, from a comparison of Panels A and B to Panels C and D, we can see that, for all methods, adjusting for covariates improves power in the settings in which covariates play a role in explaining the phenotypic variation (*θ*_*a*_ < 1 or *π*_*a*_ < .8) and does not compromise power in the other cases.

In Panels C and D, it can be seen that CERAMIC has the highest power in all settings. In contrast, GMMAT is severely underpowered, having the lowest power (or power not significantly different from the lowest) for all settings. Among the three methods that do not correct for missing data (CARAT, EMMAX and GMMAT), CARAT, which is a retrospective method, has the highest power for all the settings in which the model is misspecified (*θ*_*a*_ ≤ .8 in Panel C and *π*_*a*_ ≤ .6 in Panel D), likely reflecting the greater robustness to trait model misspecification of the retrospective methods. The power difference between the methods that correct for missing data (CERAMIC and MQLS-LOG) and the others is quite large (up to 6-fold), particularly in the settings in which covariates play an important role.

### Power comparison with complete data

S8, S9, S10 and S11 Tables give power for scenarios analogous to those in Fig 2 but with complete data instead of partially missing data. S12 and S13 Tables give power results for additional complete-data scenarios that include effects of shared environment on the trait and more stringent ascertainment on the unrelated individuals in a sample (ascertainment setting B) or more stringent ascertainment due to reduced prevalence (value .01).

With complete data, when all relevant covariates are included in the model, the three binary trait methods have approximately equal power in all scenarios. However, when relevant covariates are excluded from the model, the power of the prospective method GMMAT is lower than that of the retrospective methods CERAMIC and CARAT. The MQLS-LOG method, which has a logistic mean structure but does not include an additive polygenic VC has power approximately equal to that of the most powerful binary trait methods (CERAMIC and CARAT) when the additive polygenic variance is low, but loses power when the additive polygenic variance accounts for a high proportion of the trait variance. The EMMAX and MASTOR methods, which have an additive polygenic VC but linear instead of logistic mean structure, have power approximately equal to that of the most powerful methods (CERAMIC, CARAT and GMMAT) when covariates do not play an imporant role in the trait model and additive polygenic variance does. Compared to the retrospective method MASTOR, the prospective method EMMAX loses power when relevant covariates are omitted from the fitted model in the settings in which covariates play an important role in the trait model (*θ*_*a*_ ≤ .2 or *π*_*a*_ ≤ .2). For EMMAX, in particular, this could be explained in more detail by the fact that EMMAX assesses the variation of the test statistic based on phenotypic variance (i.e., phenotypes are treated as random in EMMAX), and failure to adjust for covariates would lead to inflation of the estimated phenotypic variance, and hence, to a reduction in power, whereas the retrospective methods such as MASTOR and CERAMIC assess variation based on genotypic variance (i.e., genotypes are treated as random), so are robust to power loss arising from misspecification of the phenotype model. Regardless of whether or not covariates are adjusted for, CERAMIC has higher power than EMMAX when covariate effects are large relative to additive polygenic effects (*θ*_*a*_ ≤ .2 or *π*_*a*_ ≤ .2), and maintains similar power to EMMAX in other scenarios.

### Analysis of T2D data from the FHS

For the analysis of T2D data from the FHS (sample size 8080 individuals with 367,770 SNPs after quality control), we restrict consideration to methods that did not experience type 1 error problems in our simulations. We compare CERAMIC, MASTOR, EMMAX, MQLS-LOG and MQLS-LIN. Tables 4 and 5 report the estimates, with standard errors, of the regression parameters and VCs obtained by CERAMIC and MASTOR (which use different null phenotypic models). The Q-Q plots (not presented) for the genome scan p-values from all five methods do not exhibit any evidence of inflation, and their genomic control inflation factors [25] are all below 1.01.

**Table 4:** Parameter Estimates, $\left({\hat{\xi }}_{0},{\hat{\beta }}_{0}\right)$, in the Null Phenotypic Model of CERAMIC, for Type 2 Diabetes in the Framingham Heart Study.

Parameter	Estimate	SE
*ξ* (VC parameter)	.41	−
Intercept	-6.3	.41
Coefficient of sex	-.75	.1
Coefficient of BMI	.24	.01

Sex is coded as female = 2, male = 1.

**Table 5:** Parameter Estimates in MASTOR’s Null Phenotypic Model for Type 2 Diabetes in the Framingham Heart Study.

Parameter	MLE (SE)
*h* (heritability)	.45 (.08)
$\sigma_{a}^{2}$ (additive variance)	.074 (.01)
$\sigma_{e}^{2}$ (environmental variance)	.090 (.01)
$\sigma_{T}^{2}$ (total variance)	.16 (.005)
Intercept	-.64 (.06)
Coefficient of sex	-.12 (.02)
Coefficient of BMI	.041 (.002)

There are only 2 independently specified VC parameters in the model; the 4 VC parameters in the table are related by the equations $h=\sigma_{a}^{2}/\sigma_{T}^{2}$ and $\sigma_{a}^{2}+\sigma_{e}^{2}=\sigma_{T}^{2}$. Sex is coded as female = 2, male = 1.

Table 6 presents the p-values for the SNPs with the strongest association signals with T2D, i.e., the SNPs for which at least one of CERAMIC, MASTOR and EMMAX gives a p-value < 2 × 10^−5^. The two SNPs with the smallest p-values, rs4506565 and rs7901695, are in an intron of *TCF7L2* (MIM 602228), which has been extensively reported to have strong association with T2D [26–28]. Among the other 5 genes listed in the table, *TLE1* (MIM 600189) has previously been reported and replicated as a T2D susceptibility locus [29, 30], and *GALNT9* (MIM 606251) is the left flanking gene of SNP rs10747083 previously found to be significantly associated with fasting glucose [31]. *DLGAP1* has previously been associated with serum insulin-like growth factor-binding protein 3 (IGFBP-3) levels [32]. *DLGAP1* has also been previously associated [33] with levels of cardiac troponin T measured by a highly sensitive assay (hs-cTnT), where hs-cTnT has been found [34] to be associated with diabetes mellitus in patients with stable coronary artery disease. *PALLD* has previously been associated [35] with aspartate aminotransferase (AST) level, where elevated AST level has shown evidence of possible association with risk of T2D [36].

**Table 6:** SNPs with Strongest Association with Type 2 Diabetes in the Framingham Heart Study.

				P-value Based on				
SNP	Chr	Position	Nearest Gene	CERAMIC	MASTOR	EMMAX	MQLS-LOG	MQLS-LIN
rs13116548	4	169874620	*PALLD*	**1.3e-05**	2.6e-05	7.8e-05	2.7e-05	5.2e-05
rs1548315	4	169841518	*PALLD*	**1.3e-05**	2.5e-05	5.8e-05	2.9e-05	5.2e-05
rs6817551	4	169829345	*PALLD*	**1.9e-05**	3.6e-05	4.7e-05	4.7e-05	8.0e-05
rs11733251	4	169881917	*CBR4*	**1.5e-05**	2.7e-05	4.7e-05	2.9e-05	4.9e-05
rs2331450	4	169901225	*CBR4*	**1.6e-05**	2.8e-05	5.9e-05	3.2e-05	5.2e-05
rs10518037	4	169899023	*CBR4*	**1.7e-05**	2.9e-05	5.9e-05	3.4e-05	5.6e-05
rs1531254	4	169881245	*CBR4*	**1.7e-05**	3.2e-05	5.6e-05	3.1e-05	5.4e-05
rs1870306	4	169923672	*CBR4*	**1.9e-05**	3.1e-05	8.9e-05	3.0e-05	4.6e-05
rs17083935	9	83326808	*TLE1*	**1.4e-05**	1.7e-05	2.3e-05	3.9e-05	5.8e-05
rs12004598	9	83354770	*TLE1*	**1.7e-05**	2.0e-05	4.9e-05	3.4e-05	5.0e-05
rs17083941	9	83327608	*TLE1*	**2.0e-05**	2.6e-05	5.7e-05	5.3e-05	8.0e-05
rs4506565	10	114756041	*TCF7L2*	1.7e-07	**4.4e-08**	1.3e-07	3.0e-07	1.0e-07
rs7901695	10	114754088	*TCF7L2*	3.1e-07	**9.2e-08**	5.4e-07	4.5e-07	1.6e-07
rs12243326	10	114788815	*TCF7L2*	1.0e-06	**4.6e-07**	3.8e-06	2.2e-06	1.2e-06
rs4132670	10	114767771	*TCF7L2*	1.3e-06	**3.1e-07**	1.8e-06	2.6e-06	8.8e-07
rs7488766	12	132665596	*GALNT9*	1.5e-05	6.3e-06	**3.4e-06**	2.6e-05	1.6e-05
rs11874767	18	3952917	*DLGAP1*	**9.5e-06**	1.8e-05	3.3e-05	3.5e-05	6.3e-05

Bold indicates the smallest p-value for each SNP. MIM numbers of genes not mentioned in the text: *PALLD* (MIM 608092), *CBR4* (No MIM number), *DLGAP1* (MIM 605445).

From Table 6, we observe that among the three tests that account for additive polygenic effects, i.e. CERAMIC, MASTOR and EMMAX, EMMAX almost always gives the largest p-values, while CERAMIC often yields the smallest p-values.

### Run times for CERAMIC

CERAMIC as well as MQLS-LOG and MQLS-LIN are implemented in the CERAMIC softare, which will be made freely available at http://www.stat.uchicago.edu/~mcpeek/software/index.html. We report run times for CERAMIC in simulations and in analysis of the FHS data set. All runs are completed using only one core (at 3.5GHz) of Intel Xeon CPU E5-2637 v3. In CERAMIC, the time-limiting step is the incorporation of missing data, which depends strongly on the sizes of the individual pedigrees making up the sample because the missing data incorporation is based only on genotype and phenotype information from the close relatives of the individuals with missing genotype. Therefore, because incorporation of missing data is the slowest step, for fixed size of the families making up the sample, the computation could be expected to scale approximately linearly in sample size. We report run times on simulated data sets with varying sample sizes, where each sample consists of 20% unrelated individuals and 80% related individuals in families of the type in Fig 1, tested at 50000 SNPs. For sample sizes of 1 × 10^3^, 2 × 10^3^, 4 × 10^3^, 6 × 10^3^, 8 × 10^3^ and 1 × 10^4^, we obtain run times of 4.9, 7.8, 16.2, 23.6, 32.8 and 39.0 minutes, respectively. These run times are plotted in S1 Fig, from which it is clear that the run time is indeed approximately linear for a fixed family size.

For the FHS data set, which contains 8080 individuals, it takes approximately 4.2 hours to perform a scan of 367,770 SNPs with phenotypic residuals computed once per genome screen, and approximately 6.1 hours if phenotypic residuals are computed separately for each SNP. This time is greater than would be needed for a sample of 8080 individuals of the type in our simulations because Framingham includes several families that each have hundreds of individuals, for whom the missing data step is much more time consuming. However, even in this case, the computation time would be expected to scale approximately linearly as the sample size increased, for a fixed family complexity. With complete data, the computations could be substantially sped up by taking a different algorithmic approach, similar to those used in LMM methods. However, such an approach is not optimal for partially missing data. In all cases, the computations are easily parallelized across tested variants.

## Discussion

For genetic association mapping of binary traits in samples with related individuals, we have developed a new method, CERAMIC, which incorporates pedigree and covariate information and effectively handles partially missing data. CERAMIC is applicable to samples that contain essentially arbitrary combinations of related and unrelated individuals. CERAMIC can be viewed as a hybrid of logistic regression and LMM approaches. Like LMM methods, CERAMIC incorporates an additive component of variance and can accommodate related individuals in a computationally feasible way. Like logistic regression methods, CERAMIC uses a logistic function to model the effects of covariates on a binary trait, and it accounts for the dependence of the variance on the mean (i.e. Bernoulli variance). As a result, CERAMIC is able to gain power, over LMM methods, for association mapping of binary traits. In addition to adjusting for covariates, CERAMIC can increase power by incorporating partially missing data. CERAMIC is based on a set of estimating equations, and we take a retrospective approach to assessment of significance of the test statistic, which provides a way to more easily incorporate partially missing data and also leads to robustness of the method to misspecification of the phenotype model. CERAMIC is implemented in freely-available software and is computationally feasible for current genome-wide association studies.

In simulations, we demonstrate that CERAMIC outperforms previously-proposed binary-trait methods GMMAT and CARAT in scenarios with partially missing data, with CERAMIC giving large increases in power over the other two methods in many scenarios. CERAMIC also outperforms GMMAT when the trait model is misspecified, with both large increases in power and also improved type 1 error control over GMMAT. When there are no missing data and the correct set of covariates is included in the fitted model, the three methods have approximately equal power. We show that the sophisticated handling of partially missing data in CERAMIC can recover a large portion of the power of complete data. In contrast, imputation of the mean genotype value for missing genotype data in GMMAT or CARAT does a poor job, recovering almost no power.

In a range of simulated scenarios with different types of trait models, various levels of relative importance of covariate effects and additive polygenic effects within a trait model, and either complete data or partially missing data, CERAMIC outperforms or performs as well as the best-performing of the other methods considered, MQLS-LOG, MQLS-LIN, MASTOR, EMMAX, GMMAT and CARAT. In addition, we have demonstrated that, when covariates play a major role in the trait model and relevant covariates are included in the fitted model, the methods that incorporate a logistic mean structure tend to perform better than those that incorporate a linear mean structure, even when the underlying trait model is not logistic, but instead follows a liability threshhold model. When additive polygenic effects play a major role in the trait model, the methods that include an additive polygenic VC tend to have higher power than the other methods. When data are partially missing among related individuals, the retrospective methods that incorporate sophisticated missing data handling (CERAMIC, MQLS-LOG, MQLS-LIN and MASTOR) boost power by exploiting information contained in partially missing data.

We apply our methods to analysis of T2D in the FHS data, where we replicate association with two previously-identified T2D susceptibility loci *TCF7L2* [26–28], and *TLE1* [29, 30]. In fact, of the 10 smallest CERAMIC p-values in our genomewide analysis, 9 occur in or near either known T2D susceptibility loci or plausible candidates (6 loci in total), verifying that CERAMIC is able to home in on the important loci in a genome scan.

In genetic association studies, it can be of interest to estimate the association parameter *γ*, for example, as a way to quantify the strength and direction of association and/or to build a predictive phenotype model. With complete data, *γ* can be estimated by $\hat{\gamma }$, where $\left(\hat{\gamma },\hat{\beta },\hat{\xi }\right)$ is the solution of the system consisting of Eqs (3), (4) and (8), with the standard error of $\hat{\gamma }$ given by the square root of the first diagonal element of Eq (14). With partially missing data, if our primary aim is to estimate *γ*, rather than to test for association, then we first need to make a careful choice of the set of individuals to be included in the estimation. We would naturally include all the individuals in *U*, the set of individuals with complete data, and we can also include a subset *S*′ ⊂ *S*, where *S*′ is a set of individuals who have non-missing phenotype and covariate information, and whose genotypes can be informatively estimated from genotyped relatives. We can then set *W*′ = *U* ∪ *S*′ and use Eq 19, with *W*′ and *S*′ substituted for *W* and *S*, to obtain the BLUP vector ${\hat{G}}_{W^{\prime }}$. Then *γ* can be estimated by solving the system of Eqs (3), (4) and (8), where all vectors and matrices are restricted to contain only those individuals in set *W*′ and where ${\hat{G}}_{W^{\prime }}$ is substituted for ***G***. In the presence of substantial amounts of missing data, the choice of the set *S*′ could potentially impact the properties of the resulting estimator of *γ*. While including in the estimation some ungenotyped individuals whose genotypes can be informatively estimated could improve the precision of the estimator by drawing information from genotyped relatives, inclusion of ungenotyped individuals on whose genotypes the data provide relatively low information could bias the estimate of *γ*. This is in contrast to the testing problem, in which including such individuals would simply provide a relatively low amount of additional power.

In deriving phenotypic residuals, we have used an estimating equation framework to estimate the regression coefficients and VCs. This framework, although built specifically for binary traits, can be generalized to traits with a general exponential family distribution, e.g., count phenotypes distributed as Poisson. For such traits, we propose a general mean and variance structure for testing the null hypothesis,

$$\text{E}\left(Y_{i}|X,G\right)=\mu_{i}\left(\beta \right)=g^{-1}\left(x_{i}^{T}\beta +G_{i}\gamma \right),$$ (28)

$${\text{Var}}_{0}\left(Y|X,G\right)=\text{diag}\left(\sqrt{V(\mu_{1})},\cdots ,\sqrt{V(\mu_{n})}\right)\Sigma \;\text{diag}\left(\sqrt{V(\mu_{1})},\cdots ,\sqrt{V(\mu_{n})}\right),$$ (29)

where *g*(*x*) and *V*(*μ*) are the link and variance functions, respectively, chosen for the given exponential-family distribution [37]. For binary traits, typical choices for *g*(*x*) and *V*(*μ*) are the logit function (i.e. log(*x*/(1 − *x*))) and Bernoulli variance *μ*(1 − *μ*), while for Poisson traits, typical choices would be *g*(*x*) = *log*(*x*) and *V*(*μ*) = *μ*. Furthermore, the correlation matrix **Σ** can be extended to include more VCs (e.g., dominance variance) by assuming $\Sigma =\xi_{1}\Phi_{1}+\cdots +\xi_{k}\Phi_{k}+(1-\sum_{1}^{k}\xi_{i})I$. A system of estimating equations can be constructed in a similar way and solved in a recursive fashion.

## Supporting Information

S1 Text Equivalence of Eqs (16) and (13) with Complete Data. (PDF)

S2 Text Equivalence of Eqs (16) and (20). (PDF)

S1 Table Empirical Type 1 Error with Shared Environment and More Stringent Ascertainment. (PDF)

S2 Table Empirical Type 1 Error with Prevalence 1%. (PDF)

S3 Table Empirical Type 1 Error of MQLS-LIN, MQLS-LOG, CERAMIC and GEMMA, with Partially Missing Data. (PDF)

S4 Table Power Comparison with Partially Missing Data, When All Relevant Covariates Are Included in the Fitted Model, Where Traits Are Generated by the Mixed-Effects Logistic Model. (PDF)

S5 Table Power Comparison with Partially Missing Data, When the Relevant Covariates Are Omitted from the Fitted Model, Where Traits Are Generated by the Mixed-Effects Logistic Model. (PDF)

S6 Table Power Comparison with Partially Missing Data, When All Relevant Covariates Are Included in the Fitted Model, Where Traits Are Generated by the Liability Threshold Model. (PDF)

S7 Table Power Comparison with Partially Missing Data, When the Relevant Covariates Are Omitted from the Fitted Model, Where Traits Are Generated by the Liability Threshold Model. (PDF)

S8 Table Power Comparison with Complete Data, When All Relevant Covariates Are Included in the Fitted Model, Where Traits Are Generated by the Mixed-Effects Logistic Model. (PDF)

S9 Table Power Comparison with Complete Data, When the Relevant Covariates Are Omitted from the Fitted Model, Where Traits Are Generated by the Mixed-Effects Logistic Model. (PDF)

S10 Table Power Comparison with Complete Data, When All Relevant Covariates Are Included in the Fitted Model, Where Traits Are Generated by the Liability Threshold Model. (PDF)

S11 Table Power Comparison with Complete Data, When the Relevant Covariates Are Omitted from the Fitted Model, Where Traits Are Generated by the Liability Threshold Model. (PDF)

S12 Table Power Comparison When Traits Are Generated by the Liability Threshold Model with Shared Environment Effect and More Stringent Ascertainment. (PDF)

S13 Table Power Comparison When Traits Are Generated by the Liability Threshold Model with Prevalence 1%. (PDF)

S1 Fig Run Time Results. (TIF)

## References

[1] Price AL, Patterson NJ, Plenge RM, Weinblatt ME, Shadick NA, Reich D. Principal components analysis corrects for stratification in genome-wide association studies. Nature Genetics. 2006;38(8):904–909. 10.1038/ng1847 16862161

[2] Chanock SJ, Hunter DJ. Genomics: when the smoke clears. Nature. 2008;452(7187):537–538. 10.1038/452537a 18385720

[3] Newman DL, Abney M, McPeek MS, Ober C, Cox NJ. The importance of genealogy in determining genetic associations with complex traits. The American Journal of Human Genetics. 2001;69(5):1146 10.1086/323659 11590549 PMC1274359

[4] Thornton T, McPeek MS. Case-control association testing with related individuals: a more powerful quasi-likelihood score test. The American Journal of Human Genetics. 2007;81(2):321–337. 10.1086/519497 17668381 PMC1950805

[5] McPeek MS. BLUP Genotype Imputation for Case-Control Association Testing with Related Individuals and Missing Data. Journal of Computational Biology. 2012;19(6):756–765. 10.1089/cmb.2012.0024 22697245 PMC3375641

[6] Whittemore AS, Halpern J, Ahsan H. Covariate adjustment in family-based association studies. Genetic Epidemiology. 2005;28(3):244–255. 10.1002/gepi.20055 15593089

[7] Laird NM, Horvath S, Xu X. Implementing a unified approach to family-based tests of association. Genetic Epidemiology. 2000;19(S1):S36–S42. 10.1002/1098-2272(2000)19:1+%3C::AID-GEPI6%3E3.3.CO;2-D 11055368

[8] Kang HM, Sul JH, Zaitlen NA, Kong S, Freimer NB, Sabatti C, et al Variance component model to account for sample structure in genome-wide association studies. Nature Genetics. 2010;42(4):348–354. 10.1038/ng.548 20208533 PMC3092069

[9] Lippert C, Listgarten J, Liu Y, Kadie CM, Davidson DI, Heckerman D. FaST linear mixed models for genome-wide association studies. Nature Methods. 2011;8(10):833–835. 10.1038/nmeth.1681 21892150

[10] Zhou X, Stephens M. Genome-wide efficient mixed-model analysis for association studies. Nature Genetics. 2012;11(4):407–409. 10.1038/ng.2310 PMC3386377 22706312

[11] Weissbrod O, Lippert C, Geiger D, Heckerman D. Accurate liability estimation improves power in ascertained case-control studies. Nature Methods. 2015;12:332–334. 10.1038/nmeth.3285 25664543

[12] Hayeck TJ, Zaitlen NA, Loh PR, Vilhjalmsson B, Pollack S, Gusev A, et al Mixed model with correction for case-control ascertainment increases association power. The American Journal of Human Genetics. 2015;96:720–730. 10.1016/j.ajhg.2015.03.004 25892111 PMC4570278

[13] Papachristou C, Ober C, Abney M. Genetic variance components estimation for binary traits using multiple related individuals. Genetic Epidemiology. 2011;35(5):291–302. 10.1002/gepi.20577 21465547 PMC3612980

[14] Stanhope SA, Abney M. GLOGS: a fast and powerful method for GWAS of binary traits with risk covariates in related populations. Bioinformatics. 2012;28(11):1553–1554. 10.1093/bioinformatics/bts190 22522135 PMC3356846

[15] Chen H, Wang C, Conomos MP, Stilp AM, Li Z, Sofer T, et al Control for population structure and relatedness for binary traits in genetic association studies via logistic mixed models. The American Journal of Human Genetics. 2016;98(4):653–666. 10.1016/j.ajhg.2016.02.012 27018471 PMC4833218

[16] Jiang D, Zhong S, McPeek MS. Retrospective Binary-Trait Association Test Elucidates Genetic Architecture of Crohn’s Disease. Am. J. Hum. Genet. 2016;98:243–255. 10.1016/j.ajhg.2015.12.012 26833331 PMC4746383

[17] Jakobsdottir J, McPeek MS. MASTOR: Mixed-Model Association Mapping of Quantitative Traits in Samples with Related Individuals. The American Journal of Human Genetics. 2013;92(5):652–666. 10.1016/j.ajhg.2013.03.014 23643379 PMC3644644

[18] Bourgain C, Hoffjan S, Nicolae R, Newman D, Steiner L, Walker K, Reynolds R, Ober C, McPeek MS. Novel case-control test in a founder population identifies P-selectin as an atopy susceptibility locus. Am. J. Hum. Genet. 2003;73:612–626. 10.1086/378208 12929084 PMC1180685

[19] Wang Z, McPeek MS. An Incomplete-Data Quasi-Likelihood Approach to Haplotype-Based Genetic Association Studies on Related Individuals. JASA. 2009;104:2151–2160. 10.1198/jasa.2009.tm08507 PMC2860453 20428335

[20] McPeek MS, Wu X, Ober C. Best Linear Unbiased Allele-Frequency Estimation in Complex Pedigrees. Biometrics. 2004;60(2):359–367. 10.1111/j.0006-341X.2004.00180.x 15180661

[21] Heyde CC. Quasi-likelihood and its application: a general approach to optimal parameter estimation. Springer Verlag; 1997.

[22] Abney M, Ober C, McPeek MS. Quantitative-trait homozygosity and association mapping and empirical genomewide significance in large, complex pedigrees: fasting serum-insulin level in the Hutterites. The American Journal of Human Genetics. 2002;70:920–934. 10.1086/339705 11880950 PMC379120

[23] Wang Z, McPeek MS. ATRIUM: testing untyped SNPs in case-control association studies with related individuals. The American Journal of Human Genetics. 2009;85(5):667–678. 10.1016/j.ajhg.2009.10.006 19913122 PMC2775837

[24] Rodriguez G, Goldman N. Improved estimation procedures for multilevel models with binary response: a case study. JRSS A. 2001;164:339–355.

[25] Devlin B, Roeder K. Genomic control for association studies. Biometrics. 1999;55(4):997–1004. 10.1111/j.0006-341X.1999.00997.x 11315092

[26] Burton PR, Clayton DG, Cardon LR, Craddock N, Deloukas P, Duncanson A, et al Genome-wide association study of 14,000 cases of seven common diseases and 3,000 shared controls. Nature. 2007;447(7145):661–678. 10.1038/nature05911 17554300 PMC2719288

[27] Zeggini E, Weedon MN, Lindgren CM, Frayling TM, Elliott KS, Lango H, et al Replication of genome-wide association signals in UK samples reveals risk loci for type 2 diabetes. Science. 2007;316(5829):1336–1341. 10.1126/science.1142364 17463249 PMC3772310

[28] Voight BF, Scott LJ, Steinthorsdottir V, Morris AP, Dina C, Welch RP, et al Twelve type 2 diabetes susceptibility loci identified through large-scale association analysis. Nature Genetics. 2010;42(7):579–589. 10.1038/ng.609 20581827 PMC3080658

[29] Morris AP, Voight BF, Teslovich TM, Ferreira T, Segre AV, Steinthorsdottir V, et al Large-scale association analysis provides insights into the genetic architecture and pathophysiology of type 2 diabetes. Nature Genetics. 2012;44(9):981–990. 10.1038/ng.2383 22885922 PMC3442244

[30] DIAbetes Genetics Replication And Meta-analysis (DIAGRAM) Consortium, Asian Genetic Epidemiology Network Type 2 Diabetes (AGEN-T2D) Consortium, South Asian Type 2 Diabetes (SAT2D) Consortium, Mexican American Type 2 Diabetes (MAT2D) Consortium, Type 2 Diabetes Genetic Exploration by Next-generation sequencing in multi-Ethnic Samples (T2D-GENES) Consortium, Mahajan A, et al Genome-wide trans-ancestry meta-analysis provides insight into the genetic architecture of type 2 diabetes susceptibility. Nature Genetics. 2014;46:234–244. 10.1038/ng.2897 24509480 PMC3969612

[31] Scott RA, Lagou V, Welch RP, Wheeler E, Montasser ME, Luan J, et al Large-scale association analyses identify new loci influencing glycemic traits and provide insight into the underlying biological pathways. Nature Genetics. 2012;44(9):991–1005. 10.1038/ng.2385 22885924 PMC3433394

[32] Comuzzie AG, Cole SA, Laston SL, Voruganti VS, Haack K, Gibbs RA, et al Novel Genetic Loci Identified for the Pathophysiology of Childhood Obesity in the Hispanic Population. PLoS One. 2012;7(12):e51954 10.1371/journal.pone.0051954 23251661 PMC3522587

[33] Yu B, Barbalic M, Brautbar A, Nambi V, Hoogeveen RC, Tang W, et al Association of Genome-Wide Variation with Highly Sensitive Cardiac Troponin-T (hs-cTnT) Levels in European- and African-Americans: A Meta-Analysis from the Atherosclerosis Risk in Communities and the Cardiovascular Health Studies. Circ Cardiovasc Genet. 2013;6:82–88. 10.1161/CIRCGENETICS.112.963058 23247143 PMC3693561

[34] Ucar H, Gur M, Seker T, Kaypakli O, Elbasan Z, Koyunsev NY, et al High-Sensitivity Cardiac Troponin T is Associated with SYNTAX Score and Diabetes Mellitus in Patients with Stable Coronary Artery Disease. J Clin Exp Cardiolog. 2013;4:263.

[35] Chalasani N, Guo X, Loomba R, Goodarzi MO, Haritunians T, Kwon S, et al Genome-Wide Association Study Identifies Variants Associated with Histologic Features of Nonalcoholic Fatty Liver Disease. Gastroenterology. 2010;139:1567–1576. 10.1053/j.gastro.2010.07.057 20708005 PMC2967576

[36] Kunutsor SK, Abbasi A, Apekey TA. Aspartate Aminotransferase—Risk Marker for Type-2 Diabetes Mellitus or Red Herring? Front Endocrinol. 2014;5:189 10.3389/fendo.2014.00189 PMC4219379 25408682

[37] McCullagh P, Nelder JA. Generalized linear model. vol. 37 Chapman & Hall/CRC; 1989.

