# Supplementary material for: CERAMIC: Case-Control Association Testing in Samples with Related Individuals, Based on Retrospective Mixed Model Analysis with Adjustment for Covariates
Source: PLoS Genet. 2016 Oct 3;12(10):e1006329. doi: 10.1371/journal.pgen.1006329 (PMC5047592; doi:10.1371/journal.pgen.1006329)
Supplement: S1 Text — (PDF) [file pgen.1006329.s001.pdf]

Supporting Information

S1 Text

Equivalence of Eqs. (16) and (13) with complete data. We show that with complete data, i.e. with $N = R = U = W$ and $S = V = \emptyset$, the CERAMIC statistic of Eq. (16) reduces to the CERAMIC$_c$ statistic of Eq. (13). In this case, $\boldsymbol{G}_R = \boldsymbol{G}_Q = \boldsymbol{G}$, $\hat{\boldsymbol{\Gamma}}_W$, $\hat{\boldsymbol{\Sigma}}_W$, $\hat{\boldsymbol{\mu}}_W$, $\hat{\boldsymbol{\beta}}_{W0}$, and $\hat{\xi}_{W0}$ are $\hat{\boldsymbol{\Gamma}}_0$, $\hat{\boldsymbol{\Sigma}}_0$, $\hat{\boldsymbol{\mu}}_0$, $\hat{\boldsymbol{\beta}}_0$, and $\hat{\xi}_0$, respectively, $\boldsymbol{Z}_W = \boldsymbol{Z}$, $\boldsymbol{\Phi}_{RW} = \boldsymbol{\Phi}_R = \boldsymbol{\Phi}$, $\boldsymbol{P}_Q = \boldsymbol{P} = \boldsymbol{M}$, $n = q$, and $\check{\sigma}_G^2 = \hat{\sigma}_G^2$. It remains to show that $\boldsymbol{F} = \boldsymbol{Z}$. To see that this holds, first recall that we obtain $(\hat{\boldsymbol{\beta}}_0, \hat{\xi}_0)$ by solving Eqs. (3) and (4) with γ set to 0, and that $\hat{\boldsymbol{\Gamma}}_0$, $\hat{\boldsymbol{\Sigma}}_0$, and $\hat{\boldsymbol{\mu}}_0$ are $\boldsymbol{\Gamma}$, $\boldsymbol{\Sigma}$, and $\boldsymbol{\mu}$, respectively, evaluated at $(\gamma, \boldsymbol{\beta}, \xi) = (0, \hat{\boldsymbol{\beta}}_0, \hat{\xi}_0)$. Then from Eq. (3), we have $\boldsymbol{X}^T\boldsymbol{Z} = \boldsymbol{X}^T\hat{\boldsymbol{\Gamma}}_0^{1/2}\hat{\boldsymbol{\Sigma}}_0^{-1}\hat{\boldsymbol{\Gamma}}_0^{-1/2}(\boldsymbol{Y} - \hat{\boldsymbol{\mu}}_0) = 0$. In particular, since the vector $\mathbf{1}_n$, of length n with every element equal to 1, is one of the columns of $\boldsymbol{X}$, this implies $\mathbf{1}_n^T\boldsymbol{Z} = 0$. Then, in the complete data case, $\boldsymbol{F} = \boldsymbol{M}\boldsymbol{\Phi}_{RW}\boldsymbol{Z}_W = \boldsymbol{P}\boldsymbol{\Phi}\boldsymbol{Z} = (\boldsymbol{I} - \boldsymbol{\Phi}^{-1}\mathbf{1}_n(\mathbf{1}_n^T\boldsymbol{\Phi}^{-1}\mathbf{1}_n)^{-1}\mathbf{1}_n^T)\boldsymbol{Z} = \boldsymbol{Z}$. From this, it is clear that Eq. (16) reduces to Eq. (13) in the complete data case.
