# Supplementary material for: CERAMIC: Case-Control Association Testing in Samples with Related Individuals, Based on Retrospective Mixed Model Analysis with Adjustment for Covariates
Source: PLoS Genet. 2016 Oct 3;12(10):e1006329. doi: 10.1371/journal.pgen.1006329 (PMC5047592; doi:10.1371/journal.pgen.1006329)
Supplement: S2 Text — (PDF) [file pgen.1006329.s002.pdf]

Supporting Information

S2 Text

Equivalence of Eqs. (16) and (20). We show that the CERAMIC statistic of Eq. (16) can be rewritten as Eq. (20), in terms of the BLUP-imputed genotypes. It is sufficient to show that $\boldsymbol{Z}_W^T \hat{\boldsymbol{G}}_W = \boldsymbol{F}^T \boldsymbol{G}_R$. By a similar argument to that in S1 Text, we have that $\mathbf{1}_w^T \boldsymbol{Z}_W = 0$. By Eq. (18), $\boldsymbol{Z}_W^T \hat{\boldsymbol{G}}_W = \boldsymbol{Z}_W^T [\mathbf{1}_w (\mathbf{1}_r^T \boldsymbol{\Phi}_R^{-1} \mathbf{1}_r)^{-1} \mathbf{1}_r^T \boldsymbol{\Phi}_R^{-1} + \boldsymbol{\Phi}_{WR} \boldsymbol{M}] \boldsymbol{G}_R$. Applying $\boldsymbol{Z}_W^T \mathbf{1}_w = 0$, we obtain $\boldsymbol{Z}_W^T \hat{\boldsymbol{G}}_W = \boldsymbol{Z}_W^T \boldsymbol{\Phi}_{WR} \boldsymbol{M} \boldsymbol{G}_R = \boldsymbol{F}^T \boldsymbol{G}_R$ by the definition of $\boldsymbol{F}$. From this, it is clear that the CERAMIC statistic of Eq. (16) can be rewritten as Eq. (20), in terms of the BLUP-imputed genotypes.
