# Supplementary material for: CERAMIC: Case-Control Association Testing in Samples with Related Individuals, Based on Retrospective Mixed Model Analysis with Adjustment for Covariates
Source: PLoS Genet. 2016 Oct 3;12(10):e1006329. doi: 10.1371/journal.pgen.1006329 (PMC5047592; doi:10.1371/journal.pgen.1006329)
Supplement: S1 Table — (PDF) [file pgen.1006329.s003.pdf]

Supporting Information

S1 Table

Empirical Type 1 Error with Shared Environment and More Stringent Ascertainment.

Complete Data or Partially Missing Data	Setting of (π_a, π_c)	MQLS-LIN	MQLS-LOG	CERAMIC
Partially Missing	$(.2, .6)$	.051	.049	.049
Partially Missing	$(.8, 0)$	.048	.048	.048
Complete	$(.2, .6)$	.050	.050	.050
Complete	$(.8, 0)$	.050	.050	.048

All tests adjust for covariates. Traits are generated by the liability threshold model, where π_a is the proportion of total liability variance that is due to polygenic effects and π_c is the proportion due to covariate effects. In each case, the shared environment effect accounts for 10% of the total liability variance, and the error variance also accounts for 10% of the total liability variance. Ascertainment setting B is used. The numbers of individuals sampled in each simulation replicate are 1200, 2000, 600, and 1000, in rows 1-4, respectively. Association is tested at a non-causal SNP, which has MAF .2, and type 1 error is assessed empirically at significance level .05. Empirical type 1 error is based on 25,000 replicates. The radius of the 95% confidence interval is .0027.
