# Supplementary material for: CERAMIC: Case-Control Association Testing in Samples with Related Individuals, Based on Retrospective Mixed Model Analysis with Adjustment for Covariates
Source: PLoS Genet. 2016 Oct 3;12(10):e1006329. doi: 10.1371/journal.pgen.1006329 (PMC5047592; doi:10.1371/journal.pgen.1006329)
Supplement: S2 Table — (PDF) [file pgen.1006329.s004.pdf]

Supporting Information

S2 Table

Empirical Type 1 Error with Prevalence 1%.

Complete Data or Partially Missing Data	Setting of (π_a, π_c)	MQLS-LIN	MQLS-LOG	CERAMIC
Partially Missing	$(.2, .6)$	.050	.051	.051
Partially Missing	$(.8, 0)$	.048	.048	.050
Complete	$(.2, .6)$	.050	.052	.051
Complete	$(.8, 0)$	.049	.049	.050

All tests adjust for covariates. Traits are generated by the liability threshold model, where π_a is the proportion of total liability variance that is due to polygenic effects and π_c is the proportion due to covariate effects. Ascertainment setting A is used. The numbers of individuals sampled in each simulation replicate are 1200 for the scenarios with partially missing data and 600 for the scenarios with complete data. Association is tested at a non-causal SNP, which has MAF .2, and type 1 error is assessed empirically at significance level .05. Empirical type 1 error is based on 25,000 replicates. The radius of the 95% confidence interval is .0027.
