# Supplementary material for: CERAMIC: Case-Control Association Testing in Samples with Related Individuals, Based on Retrospective Mixed Model Analysis with Adjustment for Covariates
Source: PLoS Genet. 2016 Oct 3;12(10):e1006329. doi: 10.1371/journal.pgen.1006329 (PMC5047592; doi:10.1371/journal.pgen.1006329)
Supplement: S3 Table — (PDF) [file pgen.1006329.s005.pdf]

Supporting Information

S3 Table

Empirical Type 1 Error of MQLS-LIN, MQLS-LOG, CERAMIC and GEMMA, with Partially Missing Data.

Setting of (π_a, π_c)	MQLS-LIN	MQLS-LOG	CERAMIC	GEMMA with genotype imputation	GEMMA without genotype imputation
$(0, .8)$	.048	.049	.049	**.059**	**.032**
$(.2, .6)$	.050	.050	.050	**.062**	**.035**
$(.4, .4)$	.051	.051	.050	**.063**	**.032**
$(.6, .2)$	.050	.050	.051	**.063**	**.030**
$(.8, 0)$	.049	.049	.050	**.055**	**.032**

All tests adjust for covariates. Traits are generated by the liability threshold model, where π_a is the proportion of total liability variance that is due to polygenic effects and π_c is the proportion due to covariate effects. Ascertainment setting A is used. The number of individuals sampled in each simulation replicate is 1200. Association is tested at a non-causal SNP, which has MAF .2, and type 1 error is assessed empirically at significance level .05. Empirical type 1 error is based on 25,000 replicates. The radius of the 95% confidence interval is .0027. Bold indicates a type 1 error rate that is outside the 95% confidence interval.
