# Supplementary material for: CERAMIC: Case-Control Association Testing in Samples with Related Individuals, Based on Retrospective Mixed Model Analysis with Adjustment for Covariates
Source: PLoS Genet. 2016 Oct 3;12(10):e1006329. doi: 10.1371/journal.pgen.1006329 (PMC5047592; doi:10.1371/journal.pgen.1006329)
Supplement: S8 Table — (PDF) [file pgen.1006329.s010.pdf]

Supporting Information

S8 Table

Power Comparison with Complete Data, When All Relevant Covariates are Included in the Fitted Model, Where Traits are Generated by the Mixed-Effects Logistic Model.

**Setting of** (θ_a, θ_c)	**MQLS-LIN**	**MQLS-LOG**	**CERAMIC**	**GMMAT**	**EMMAX**	**MASTOR**	**CARAT**
$(0, .1)$	.68	**.95**	**.94**	**.95**	.67	.68	**.95**
$(.2, .8)$	.55	**.64**	.63	**.65**	.54	.54	.63
$(.4, .6)$	.45	.47	.47	**.49**	.46	.45	**.48**
$(.6, .4)$	.37	.38	**.39**	**.40**	**.40**	**.39**	**.39**
$(.8, .2)$	.31	.31	**.34**	.33	**.35**	**.35**	**.35**
$(1, 0)$	.26	.26	**.29**	.27	**.29**	**.29**	**.29**

Bold indicates the highest estimated power, or estimated power not significantly different from the highest, for each setting. In each case, the sample size is 600 individuals, and ascertainment setting A is used. Association is tested at causal SNP 2, which has MAF .2, and power is assessed empirically at significance level .05, based on 10,000 replicates. An upper bound (obtained based on power 0.5) for the standard error of each power estimate is 0.005. For a pairwise comparison, the upper bound is ∼0.007. θ_a is the ratio of the variance explained by polygenic effects to the total variance of polygenic and covariate effects on the logit scale, and $\theta_c = 1 - \theta_a$.
