# Supplementary material for: CERAMIC: Case-Control Association Testing in Samples with Related Individuals, Based on Retrospective Mixed Model Analysis with Adjustment for Covariates
Source: PLoS Genet. 2016 Oct 3;12(10):e1006329. doi: 10.1371/journal.pgen.1006329 (PMC5047592; doi:10.1371/journal.pgen.1006329)
Supplement: S10 Table — (PDF) [file pgen.1006329.s012.pdf]

Supporting Information

S10 Table

Power Comparison with Complete Data, When All Relevant Covariates are Included in the Fitted Model, Where Traits are Generated by the Liability Threshold Model.

**Setting of** (π_a, π_c)	**MQLS-LIN**	**MQLS-LOG**	**CERAMIC**	**GMMAT**	**EMMAX**	**MASTOR**	**CARAT**
$(0, .8)$	.64	**.77**	**.76**	**.77**	.62	.64	.75
$(.2, .6)$	.54	**.58**	**.57**	**.58**	.54	.54	.56
$(.4, .4)$	.44	**.45**	**.45**	**.46**	**.45**	.44	**.45**
$(.6, .2)$	.35	.35	**.36**	**.36**	**.37**	**.37**	**.37**
$(.8, 0)$	.29	.29	**.31**	**.30**	**.31**	**.31**	**.31**

Bold indicates the highest estimated power, or estimated power not significantly different from the highest, for each setting. In each case, the sample size is 600 individuals, and ascertainment setting A is used. Association is tested at causal SNP 2, which has MAF .2, and power is assessed empirically at significance level .05, based on 10,000 replicates. An upper bound (obtained based on power 0.5) for the standard error of each power estimate is 0.005. For a pairwise comparison, the upper bound is ∼0.007. π_a is the proportion of total liability variance that is due to polygenic effects and π_c is the proportion due to covariate effects.
