# Supplementary material for: CERAMIC: Case-Control Association Testing in Samples with Related Individuals, Based on Retrospective Mixed Model Analysis with Adjustment for Covariates
Source: PLoS Genet. 2016 Oct 3;12(10):e1006329. doi: 10.1371/journal.pgen.1006329 (PMC5047592; doi:10.1371/journal.pgen.1006329)
Supplement: S12 Table — (PDF) [file pgen.1006329.s014.pdf]

Supporting Information

S12 Table

Power Comparison When Traits are Generated by the Liability Threshold Model with Shared Environment Effect and More Stringent Ascertainment.

Setting of (π_a, π_c)	With missing data	MQLS-LIN	MQLS-LOG	CERAMIC	GMMAT	EMMAX	MASTOR	CARAT
(.2, .6)	YES	.54	**.59**	**.60**	.34	.28	.55	.37
	NO	.53	**.57**	**.56**	**.57**	.52	.53	**.56**
(.8, 0)	YES	.33	.33	**.39**	.22	.31	**.40**	.28
	NO	.47	.47	**.51**	.49	**.52**	**.52**	**.52**

Bold indicates the highest estimated power, or estimated power not significantly different from the highest, for each setting. All scenarios have 10% shared environment variance for siblings and 10% i.i.d error variance, and they all use ascertainment setting B, i.e., 1:1 case-control ascertainment for unrelated individuals in addition to the phenotype-based ascertainment for families. For the setting $(\pi_a, \pi_c) = (.2, .6)$, 60 families and 240 unrelated individuals (1200 individuals in total) are used in the simulation with incomplete data, while 30 families and 120 unrelated individuals (600 individuals in total) are used in the simulation with complete data. For the setting $(\pi_a, \pi_c) = (.8, 0)$, 100 families and 400 unrelated individuals (2000 individuals in total) are used in the simulation with incomplete data, while 50 families and 200 unrelated individuals (1000 individuals in total) are used in the simulation with complete data. In each case, association is tested at causal SNP 2, which has MAF .2, and power is assessed empirically at significance level .05, based on 10,000 replicates. An upper bound (obtained based on power 0.5) for the standard error of each power estimate is 0.005. For a pairwise comparison, the upper bound is $\sim$0.007. π_a is the proportion of total liability variance that is due to polygenic effects and π_c is the proportion due to covariate effects.
