# Supplementary material for: CERAMIC: Case-Control Association Testing in Samples with Related Individuals, Based on Retrospective Mixed Model Analysis with Adjustment for Covariates
Source: PLoS Genet. 2016 Oct 3;12(10):e1006329. doi: 10.1371/journal.pgen.1006329 (PMC5047592; doi:10.1371/journal.pgen.1006329)
Supplement: S13 Table — (PDF) [file pgen.1006329.s015.pdf]

Supporting Information

S13 Table

Power Comparison When Traits are Generated by the Liability Threshold Model with Prevalence 1%.

**Setting of** (π_a, π_c)	**With missing data**	**MQLS-LIN**	**MQLS-LOG**	**CERAMIC**	**GMMAT**	**EMMAX**	**MASTOR**	**CARAT**
(.2, .6)	YES	**.75**	**.76**	**.76**	.34	.28	**.75**	.32
	NO	.57	**.59**	**.58**	**.59**	.56	.56	.57
(.8, 0)	YES	.38	.38	**.39**	.16	.23	**.40**	.19
	NO	.30	.30	**.31**	**.31**	**.32**	**.31**	**.32**

Bold indicates the highest estimated power, or estimated power not significantly different from the highest, for each setting. For the missing data scenarios, 60 families and 240 unrelated individuals (1200 individuals in total) are used in the simulation, while 30 families and 120 unrelated individuals (600 individuals in total) are used in the scenarios with no missing data. Ascertainment setting A is used. In each case, association is tested at causal SNP 2, which has MAF .2, and power is assessed empirically at significance level .05, based on 10,000 replicates. An upper bound (obtained based on power 0.5) for the standard error of each power estimate is 0.005. For a pairwise comparison, the upper bound is ∼0.007. π_a is the proportion of total liability variance that is due to polygenic effects and π_c is the proportion due to covariate effects.
