# Supplementary figures and images for: CERAMIC: Case-Control Association Testing in Samples with Related Individuals, Based on Retrospective Mixed Model Analysis with Adjustment for Covariates
Source: PLoS Genet. 2016 Oct 3;12(10):e1006329. doi: 10.1371/journal.pgen.1006329 (PMC5047592; doi:10.1371/journal.pgen.1006329)

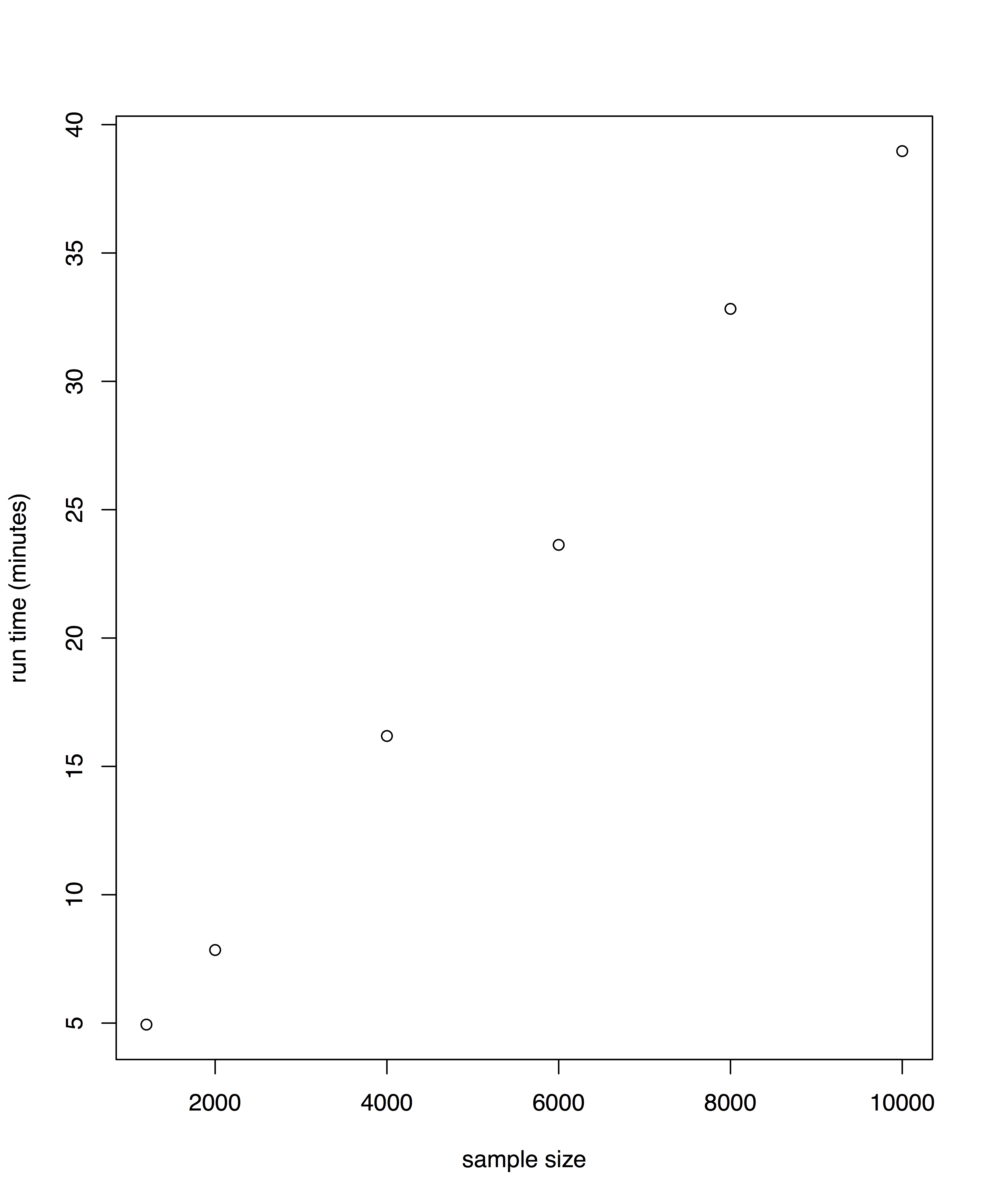

Supplement: S1 Fig — (TIF) [file pgen.1006329.s016.tif]
